# Structural basis of prokaryotic ubiquitin-like protein engagement and translocation by the mycobacterial Mpa-proteasome complex

**DOI:** 10.1038/s41467-021-27787-3

**Published:** 2022-01-12

**Authors:** Mikhail Kavalchuk, Ahmad Jomaa, Andreas U. Müller, Eilika Weber-Ban

**Affiliations:** ETH Zurich, Institute of Molecular Biology & Biophysics, CH-8093 Zurich, Switzerland

**Keywords:** Proteasome, Cryoelectron microscopy, Bacterial structural biology

## Abstract

Proteasomes are present in eukaryotes, archaea and Actinobacteria, including the human pathogen *Mycobacterium tuberculosis*, where proteasomal degradation supports persistence inside the host. In mycobacteria and other members of Actinobacteria, prokaryotic ubiquitin-like protein (Pup) serves as a degradation tag post-translationally conjugated to target proteins for their recruitment to the mycobacterial proteasome ATPase (Mpa). Here, we use single-particle cryo-electron microscopy to determine the structure of Mpa in complex with the 20S core particle at an early stage of pupylated substrate recruitment, shedding light on the mechanism of substrate translocation. Two conformational states of Mpa show how substrate is translocated stepwise towards the degradation chamber of the proteasome core particle. We also demonstrate, in vitro and in vivo, the importance of a structural feature in Mpa that allows formation of alternating charge-complementary interactions with the proteasome resulting in radial, rail-guided movements during the ATPase conformational cycle.

## Abstract (web summary)

Pup is the bacterial analog of ubiquitin for targeting proteins to the proteasome. Here, the authors use cryoEM to visualize structures of the *Mycobacterium tuberculosis* proteasome translocating a Pup-tagged substrate.

## Introduction

Energy-dependent protein degradation contributes to cellular protein quality control and general protein turnover, and ensures a dynamic proteome response to changing cellular states and environments^1,2^. It is carried out by compartmentalizing degradation machines consisting of a proteolytic core cylinder that must associate with ring-shaped, ATP-dependent regulator complexes in order to recruit, unfold and translocate its target protein substrates for processive degradation^3^. In eukaryotes, this activity is carried out by the 26S proteasome made of the 20S core particle (CP) and the 19S regulatory particle, which recruits proteins post-translationally modified with polyubiquitin (poly-Ub) chains^4^. Bacteria usually do not have proteasomes but possess complexes of related, modular architecture such as the Clp–protease complexes and HslUV. Interestingly, mycobacteria and other members of Actinobacteria have in addition obtained 20S proteasome subunits by horizontal gene transfer^5,6^. The bacterial 20S proteasome interacts with a ring-shaped AAA regulatory complex called the mycobacterial proteasome ATPase (Mpa) in mycobacteria or ARC (ATPase forming ring-shaped complexes) in other actinobacterial species^7–10^. Furthermore, these bacteria evolved a unique substrate tagging and proteasome delivery pathway that is functionally analogous to ubiquitination^11,12^. The prokaryotic ubiquitin-like protein Pup is post-translationally ligated to lysine side chains of substrate proteins by the Pup ligase PafA, which marks them for degradation by the Mpa–20S CP complex^13,14^. The resulting isopeptide linkage is formed between the side-chain carboxylate of the C-terminal glutamate residue of Pup and the substrate lysine^15^. Pupylation can be reversed by the depupylase Dop that catalyzes the cleavage of the isopeptide bond between Pup and substrate proteins^16,17^. Unlike ubiquitin, Pup is intrinsically disordered in its free state^18–20^ or when covalently attached to substrate proteins^21^. However, upon binding to the pupylation/depupylation enzymes, it forms two orthogonal helices in the C-terminal half of its primary sequence, fitting closely into the 40–50 Å-long Pup-binding groove present on Dop and PafA^22^. Pupylation and proteasomal degradation were shown to ensure survival of the bacteria under challenging conditions like during persistence of *Mycobacterium tuberculosis* (Mtb) inside host macrophages, under nitrogen-starvation or under DNA-damaging conditions^23–27^.

Mpa/ARC is related in architecture and domain structure to the six eukaryotic proteasomal ATPase subunits Rpt1-6 present in the 19S regulatory particle^9^. The protomer features the canonical AAA module, preceded by two consecutive β-barrel-shaped oligonucleotide-binding (OB) domains and an N-terminal α-helical domain^28^. In the hexameric ring, the N-terminal helices form a total of three coiled-coils by interacting with their respective neighboring N-terminal α-helical domains. The three coiled-coils emanate from the stiff flange-like structure formed by the six double-OB domains. While in the eukaryotic 19S regulatory particle the coiled-coil domains mediate binding of non-ATPase subunits (Rpn) and the positioning of ubiquitin receptors^29,30^, they constitute the direct substrate recruitment domains in Mpa/ARC^19^. Pup undergoes another disorder-to-order transition, when binding to the Mpa coiled-coil domains, forming a single long helix of 30 residues (21–51 in Mtb) that engages into an antiparallel shared coiled-coil interaction with the Mpa N-terminal coiled-coil domains^19,31^. Due to the antiparallel arrangement of the interaction with Mpa, the N-terminal region of Pup points toward the Mpa pore. Interestingly, the N-terminal segment of Pup remains disordered and acts as a threader sequence for Mpa pore engagement^8,32^. Subsequent unfolding and translocation is mediated by Mpa pore loops carrying the canonical Ar-Hb-Gly motif (F341-V342-G343 in Mtb) conserved in AAA family members^8^.

Binding of Mpa to the proteasome core is mediated via a C-terminal GQYL motif, featuring a penultimate tyrosine^8,33^, similar to the eukaryotic 19S ATPase subunits that employ the conserved C-terminal HbYX motif^34,35^. Stable interaction and consequently efficient degradation of pupylated substrates by the Mpa–proteasome of Mtb in vitro could only be observed with an open-gate variant lacking the N-terminal seven residues on the α-subunits, even though the full-length core particle is active in vivo^36^. An X-ray structure of the Mpa hexamer in the absence of the 20S proteasome and without substrate suggested that due to a stable β-grasp domain preceding the C-terminal proteasome-interaction motif, the C-termini are located inside the Mpa channel and are unavailable for interaction with the proteasome^28^. However, cryo-electron microscopic analysis of a partially resolved Mpa-hexamer suggested that this region might show some conformational flexibility^37^.

Despite the importance of the Mtb proteasome as a virulence factor for one of the most successful human pathogens^27,38,39^, the structural and biochemical information on its interaction with the AAA unfoldase Mpa, responsible for recruitment of pupylated substrates, is still limited.

Here, we utilize single-particle cryo-electron microscopy (cryo-EM) to determine the structure of the Mpa–proteasome complex at the stage of Pup translocation into the Mpa pore. We resolved two distinct conformational states corresponding to different stages during substrate translocation by the hexameric ATPase ring. The spiral-staircase arrangement of the Mpa pore loops around the translocating substrate chain is in agreement with the canonical AAA+ substrate unfolding mechanism. Interestingly, our structure suggests that, in spite of considerable changes in the Mpa conformation taking place during substrate translocation, Mpa interactions with the proteasome are maintained through the formation of a series of charge-complementary contacts along radially distributed rail-like features of the proteasome core particle. The importance of these interactions for the Mpa–proteasome substrate translocation/degradation mechanism is further supported by in vitro biochemical data as well as in vivo substrate degradation.

## Results

### Generation of a substrate-engaged Mpa–proteasome complex for structural analysis

**Fig. 1:**
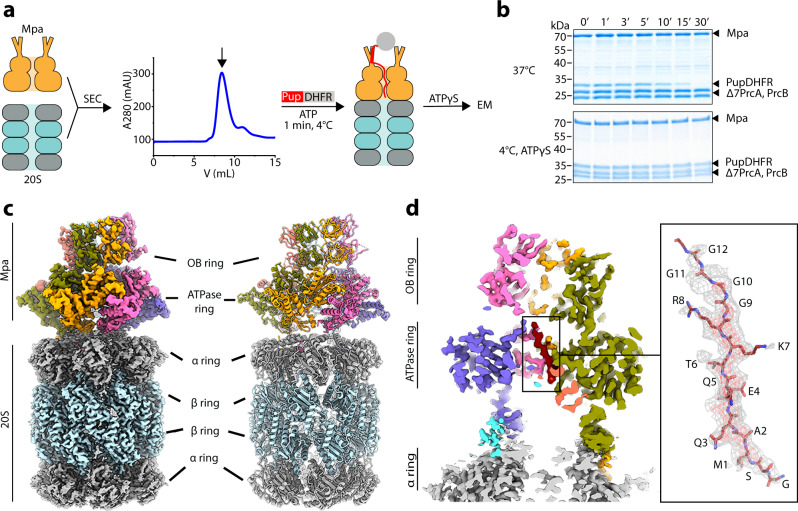
Generation and structure determination of a substrate-engaged Mpa–20S proteasome complex from *Mycobacterium tuberculosis*. **a** Cartoon depicting generation of the stalled, substrate-engaged Mpa–20S complex. Open-gate 20S core particle (gray and light blue), Mpa (orange) and ATP were incubated together and the mixture was subjected to size-exclusion chromatography (SEC; the SEC profile image was generated using Origin 2018). The Mpa–20S core particle elution peak, pupylated model substrate (linear Pup—dihydrofolate reductase fusion PupDHFR) and ATP were incubated for 60 s on ice. After addition of ATPγS, the sample was immediately applied onto the EM grid. **b** PupDHFR degradation was monitored by Coomassie-stained SDS-PAGE. The degradation reaction containing open-gate proteasome (Δ7PrcA, PrcB), wild-type Mpa and PupDHFR was started by addition of ATP and was carried out at 37 °C (upper gel) and at 4 °C with the addition of ATPγS after 1 min (lower gel). Aliquots for SDS-PAGE analysis were taken at the indicated time points. Representatives of three individual experiments are shown. Source Data are provided as a Source Data file. **c** Composite cryo-EM map of the 20S proteasome and substrate-engaged Mpa in state A after 3D image classification and focused 3D refinement. Cryo-EM densities of 20S α and β subunits are colored gray and light blue, respectively. Mpa protomes densities representing oligonucleotide-binding (OB) and ATPase domains are colored pink, blue, cyan, yellow, orange, and green. Pup is colored red. **d** Cross-section of the cryo-EM density of the 20S α-ring gate (gray) and Mpa engaged with the N-terminal Pup segment inside the AAA chamber. EM density corresponding to the first 12 residues of Pup inside the Mpa ATPase chamber is shown with fitted atomic coordinates.

In order to obtain a molecular understanding of the interactions between Mpa and the 20S proteasome as well as mechanistic insights into the engagement of pupylated substrates into the Mpa pore, we aimed to generate a substrate-engaged Mpa–20S CP complex for study by cryo-EM. To favor complex formation, seven residues were N-terminally deleted from the proteasomal α-subunits (Δ7PrcA), as this was shown to stabilize the interaction^7^. A homogeneously Pup-decorated model substrate was generated by placing the Pup coding sequence in-frame upstream of the gene *folA* encoding dihydrofolate reductase (DHFR), resulting in a linear PupDHFR fusion, in which the Pup C-terminal residue is ligated to the DHFR amino terminus through a regular peptide bond (Fig. 1a). It has been demonstrated previously that linear Pup-substrate fusions can be recruited and degraded by the Mpa–proteasome complex^8^. As expected, PupDHFR is degraded by the Mpa–20S CP complex in the presence of ATP. Under the chosen conditions at 37 °C, PupDHFR was fully degraded within 30 min after adding ATP (Fig. 1b, upper gel). When the reaction is carried out at 4 °C with the addition of ATPγS after 1 min of incubation, no discernable amount of PupDHFR is degraded in the same time frame (Fig. 1b, lower gel), suggesting that substrate is stalled at the stage of Mpa translocation, analogously to what was observed for the human 26S proteasome engaged with a ubiquitinated substrate^40^. Therefore, to prepare the sample for electron microscopy, we assembled the Mpa-20S complex in the presence of ATP, and added the pupylated substrate while keeping the sample on ice to allow for substrate translocation and slow ATP turnover. The reaction was quenched with ATPγS to prevent further cycles of ATP hydrolysis and to stall substrate translocation, before application to the electron microscopy grid and vitrification (Fig. 1a)^40^.

### Overall architecture of the substrate-engaged Mpa–proteasome complex reveals two Mpa states

We originally picked 860,718 particles for 2D classification, presenting both top and side views of the proteasome, of which 385,394 were used for 3D classification (Supplementary Fig. 1). The initial 2D and 3D image processing scheme visualized a class with Mpa-bound proteasome in addition to a class containing uncapped 20S CP. The structure of Mpa bound to the 20S CP that was determined without applying any symmetry, revealed a 6-edged, star-shaped density originating from a hexameric Mpa bound to the heptameric α-ring of the 20S CP. To improve the local resolution of Mpa, we performed local 3D classification and 3D refinement combined with signal subtraction of the 20S CP (see “Methods”). This approach resolved two states of Mpa at an average resolution of 3.8 Å and 3.9 Å, respectively (Supplementary Fig. 2). The two states correspond to two distinct motor conformations stalled at sequential stages of substrate translocation. The structure of the 20S CP particle was determined by focused refinement using a binary mask for this region and was resolved at 2.8 Å resolution (Supplementary Fig. 1, Supplementary Fig. 2, Supplementary Table 1).

**Fig. 2:**
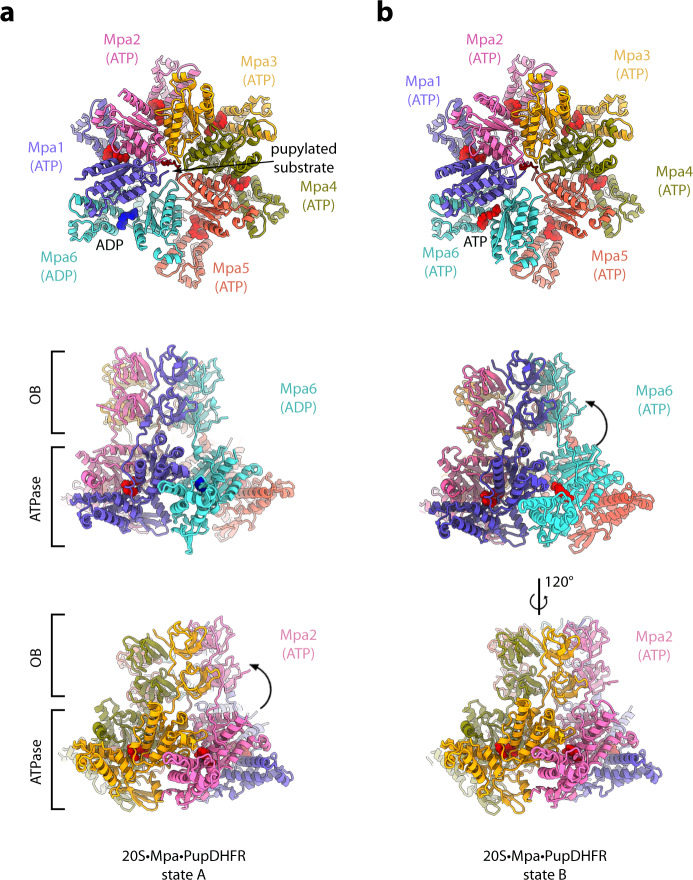
Mycobacterial AAA unfoldase Mpa engaged with PupDHFR substrate presents in two conformational states. **a**, **b** Coordinates of Mpa in state A and B are shown in cartoon representation in top view (upper) and side view (middle and lower). ATP and ADP molecules are shown as spheres and are colored red and blue, respectively. Mpa protomers (Mpa1–6) and substrate are colored with the same color code as used in Fig. 1. The conformational change in the OB-ring is indicated by an arrow.

The cryo-EM structures of the Mpa–20S CP complex reveal the architecture of a co-axially aligned AAA-protease assembly featuring a substrate conduit from the entrance pores of the slightly tilted Mpa tandem OB domains to the AAA motor and into the α-ring of the 20S CP (Fig. 1c, d). The protomers of the Mpa hexamer assemble in a spiral staircase, an arrangement that has emerged as a hallmark of AAA translocation motors^41^ (Fig. 1d; Supplementary Fig. 3). States A and B exhibit several differences including a varying degree of upward rotation (~30°) of the double-OB ring with respect to the AAA motor (Fig. 2, side views). A long loop serves as a flexible connection that can dynamically accommodate the tilt and movements in the AAA motor during substrate processing. In state A, the largest rotation was resolved at protomer 2 (pink) site, while in state B, the double-OB-ring displays the largest tilt at protomer 6 (cyan) site.

Inside the ATPase chamber of the Mpa–20S CP complex in both states, a tubular density is resolved engaged with the ATPase pore loops of the Mpa protomers (Fig. 1d; Supplementary Fig. 3). Based on the visible side-chain density, this tubular EM-density can be assigned to the first 15 residues of the Mtb Pup sequence (Fig. 1d). An additional Ser-Gly motif N-terminal of the first Pup residue is left over after TEV protease cleavage and is also resolved in the Mpa channel. The EM density of the engaged substrate becomes weaker following the Pup sequence out of the AAA chamber toward the double-OB ring of Mpa. The amino acid sequence of this region of Pup features five consecutive glycines and four aspartates, the latter usually lacking side-chain density in cryo-EM maps^42^. Substrate could not be further visualized beyond the OB rings, indicating greater conformational heterogeneity of Pup in that region. The coiled-coil N-terminal helices of Mpa that emanate from the surface of the OB ring and first recognize pupylated substrates^19,31^ are expected to be flexible and were not visualized. On the side of the proteasome, the substrate density approaches the open gate but it does not yet enter the degradation chamber (approximately 35 Å distance from the first resolved residue to the gate). Therefore, the complexes trapped in our study reveal the initial events of substrate engagement by the AAA motor prior to translocation into the proteasome for degradation.

### Mpa associates with the 20S proteasome via its C-terminal GQYL motif and the β-hairpin loops of the β-grasp domains

**Fig. 3:**
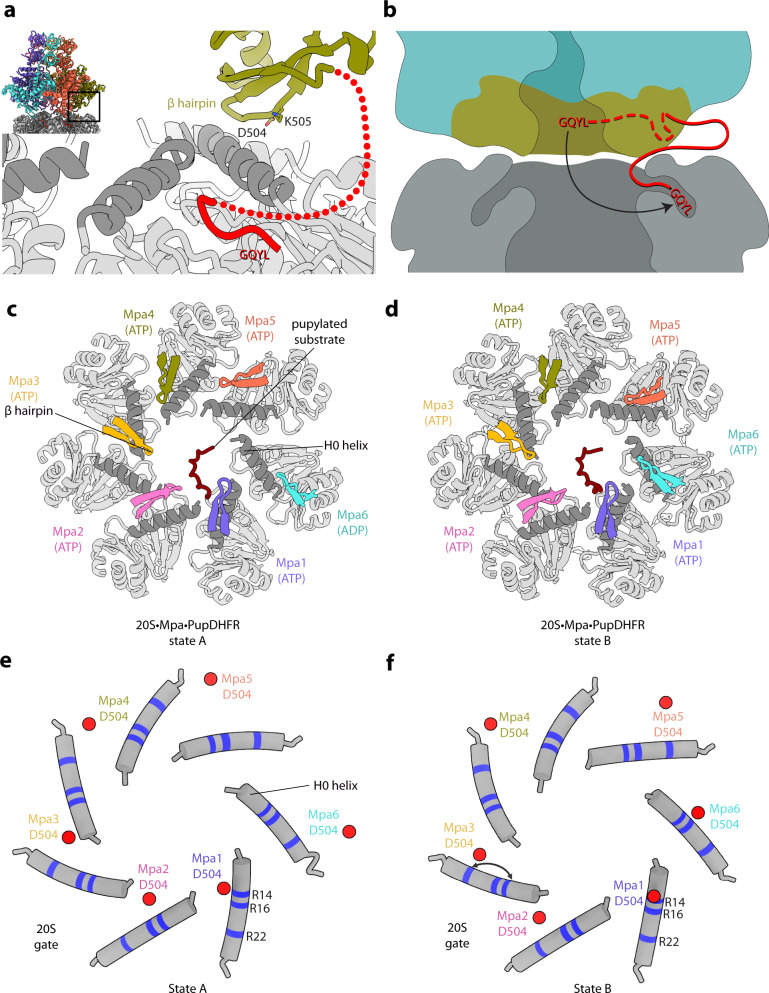
Interaction of Mpa with the proteasome α-rings. **a** Close-up of the Mpa/20S α-ring interface including the C-terminal extension of one Mpa protomer with the GQYL proteasome-interaction motif. H0 helices are colored dark gray. The dashed red line indicates the flexible fragment connecting the Mpa β-grasp domain with the GQYL C-terminal motif. Charged β-hairpin residues D504 and K505 are shown as sticks and labeled. **b** The Mpa β-grasp domain (olive) adopts a different conformation upon binding to the proteasome (gray), releasing the C-terminal GQYL motif (red) and allowing it to bind into the α-ring binding pockets (dark gray). **c** Close-up top view of the proteasome gate shown as cartoon and colored in light gray. The N-terminal H0 helices of the proteasome α-ring protomers are colored darker gray. The β-hairpin loops of Mpa (state A) are colored according to the color scheme used in Fig. 2. **d** Same as (**c**) except that the conformations of the β-hairpin loops for each of the Mpa protomers are shown as observed in state B. **e**, **f** Schematic representation of the proteasome N-terminal H0 helices shown as gray cylinders and β-hairpin loop residue D504 shown as a red dot. Charge-complementary contact residues (R14, R16, R22) on the proteasome H0 helices are marked as blue stripes. The sliding of the contacting residue (D504) in a rail-like motion on the N-terminal helices of the 20S proteasome gate is indicated by an arrow.

Proteasome regulatory ATPases are known to feature a C-terminal motif with a penultimate tyrosine residue as the main binding determinant for association with the 20S core particles to form the proteasome holocomplexes in both eukaryotes and bacteria^8,35^. A structure of Mpa determined in the absence of the proteasome showed the C-termini pointing back into the pore, presumably unavailable for interaction, although the GQYL motif itself was not resolved^28^. Our structure of the substrate-engaged proteasome resolves the interaction between Mpa and the gate of the 20S CP (Fig. 3a). It demonstrates that the C-terminal extensions of Mpa containing the terminal GQYL motifs are able to adopt a conformation facing outside of the Mpa pore, and that their GQYL motifs occupy the binding pockets between the proteasome α-ring subunits upon complex formation (Fig. 3b and Supplementary Fig. 4). Although the α-ring subunits lack the N-terminal seven residues in the open-gate 20S CP, we still observed a slight increase in proteasomal gate diameter on the Mpa-bound side compared to the Mpa-free side, which is indicative of the conformational change undergone by the proteasomal α-rings upon insertion of the GQYL motifs into the α-ring binding pockets (Supplementary Fig. 5a, b). Densities for the GQYL motif were resolved in all seven pockets due to averaging resulting from the inherent D7 symmetry of the 20S CP (Supplementary Fig. 4). We tentatively assigned six GQYL motifs based on their proximity to the respective Mpa protomers. The last GQYL motif was not assigned to any of the Mpa protomers and was included as a separate chain in the model.

Interestingly, our structure reveals that the β-grasp domains of Mpa contribute a second contact in the form of the β-hairpin loop between strands 16 and 17, which interacts with the N-terminal H0 helices that form the gate of the CP (Fig. 3a, c, d). These β-hairpin loops possess two charged (D504 and K505) residues, while the N-terminal H0 helices of the core particle α-subunits possess several glutamate (E10, E15, E18) and arginine (R14, R16, R22) residues, resulting in multiple points of possible electrostatic interactions for the hairpins along the helices. The array of charged residues along the H0 helices could serve as a “rail” for the β-hairpin loops that could move along the length of the H0 helices by forming different combinations of transient electrostatic interactions based on proximity assessment (Fig. 3e, f). In state A, Mpa protomer 6 (cyan) depicts the β-hairpin conformation that is the farthest away from the gate pore in the outward position, while protomer 1 (blue) displays the most inward position right above the pore of the CP gate (Fig. 3c, e; Supplementary Fig. 5c). These positions are slightly offset in state B (Fig. 3d, f; Supplementary Fig. 5d). While these interactions alone are not sufficient for the formation of a stable complex^8^, they likely serve to align the substrate conduit of Mpa with the gate of the CP despite the symmetry mismatch and the nucleotide-state-dependent conformational dynamics of the AAA motor.

**Fig. 4:**
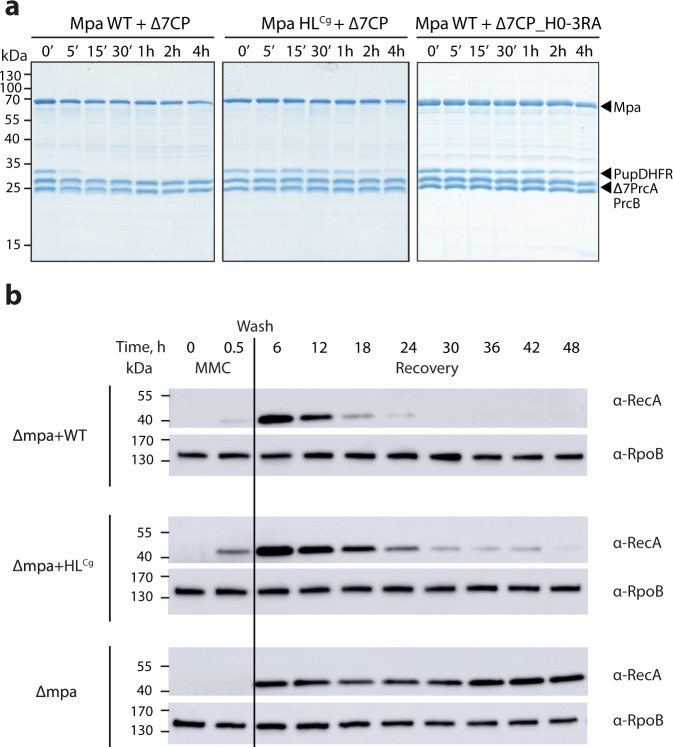
Mutation of the Mpa β-hairpin loop affects substrate degradation in vivo and in vitro. **a** Proteasomal degradation of PupDHFR mediated by wild-type Mpa (left) or Mpa HL^Cg^ (middle) in complex with open-gate 20S CP (Δ7CP) and of wild-type Mpa in complex with proteasome variant Δ7CP_H0-3RA (right) was monitored by Coomassie-stained SDS-PAGE. The reaction was started with ATP and carried out at 37 °C. Aliquots were taken at the indicated time points and analyzed by SDS-PAGE. Representatives of three individual experiments are shown. Source data are provided as a Source Data file. **b** Time-resolved degradation of RecA during the recovery after mitomycin C (MMC)-induced DNA damage stress demonstrates the effect of a β-hairpin loop mutation of Mpa in *M. smegmatis*. RecA levels were monitored upon induction of MMC stress (80 ng/mL for 30 min) and during the recovery phase in the *M. smegmatis* Δ*mpa* strain complemented with either WT Mpa or Mpa HL^Cg^. To remove MMC, cells were washed (“wash”) and then further incubated. RecA levels were analyzed by immunoblotting at the indicated time points. RpoB was probed in parallel as loading control. Representatives of three individual experiments are shown. Source Data are provided as a Source Data file.

To investigate how the β-hairpin loop interactions affect substrate degradation, an Mpa variant (Mpa HL^Cg^) was generated replacing hairpin residues 501–505 (ANGDK) with the equivalent but not conserved sequence stretch (IDGSV) from *Corynebacterium glutamicum* Mpa, where these interactions are absent, since *C. glutamicum* belongs to the group of Actinobacteria lacking the 20S CP (Supplementary Fig. 6). Additionally, a proteasome variant (H0-3RA) was generated where R14, R16 and R22 of the H0 helix were replaced with alanines. Proteasomal degradation of the model substrate PupDHFR mediated by either wild-type Mpa or the Mpa HL^Cg^ variant in complex with open-gate 20S CP (Δ7CP) as well as by wild-type Mpa in complex with the proteasome variant Δ7CP_H0-3RA was monitored by Coomassie-stained SDS-polyacrylamide gel electrophoresis (Fig. 4a). While in the presence of wild-type Mpa, PupDHFR was completely degraded within 15 min under the chosen conditions, Mpa HL^Cg^ still had not completed the reaction in four hours, indicating more than an order of magnitude change in the degradation rate. A similar effect on PupDHFR degradation was observed in the presence of wild-type Mpa in complex with the proteasome variant Δ7CP_H0-3RA.To ensure that Mpa HL^Cg^ has no defect in ATPase activity, ATP hydrolysis was followed spectroscopically by coupling the production of inorganic phosphate to the turnover of 7-methylinosine to hypoxanthine by the enzyme purine nucleotide phosphorylase. No reduction in ATP hydrolysis rate but rather an increase was observed for Mpa HL^Cg^ (237 ± 6 min^−1^) compared to Mpa WT (139 ± 1 min^−1^), excluding the possibility that the impaired ability to deliver substrates to the CP for degradation stems from a defect in ATPase activity.

To assess the effect of the β-hairpin loop in vivo, we generated a *Mycobacterium smegmatis mpa* knockout strain (Δ*mpa*) and complemented it with either wild-type Mpa or the β-hairpin variant expressed under control of the natural *mpa* promoter. The strains were transiently exposed to the DNA-damaging reagent mitomycin C, resulting first in upregulation of recombinase A (RecA), a natural substrate of the Pup-proteasome system, followed by proteasomal degradation during the recovery phase from DNA damage stress^25^. RecA immunoblotting showed that the Δ*mpa* strain complemented with the Mpa HL^Cg^ β-hairpin loop variant exhibited slower RecA degradation compared to the strain complemented with the wild-type protein (Fig. 4b). Taken together, our findings support a role of the β-hairpin loop in mediating dynamic contacts between Mpa and the CP during pupylated substrate degradation.

### Substrate is arrested in Mpa in the pore-engaged pre-handover state

**Fig. 5:**
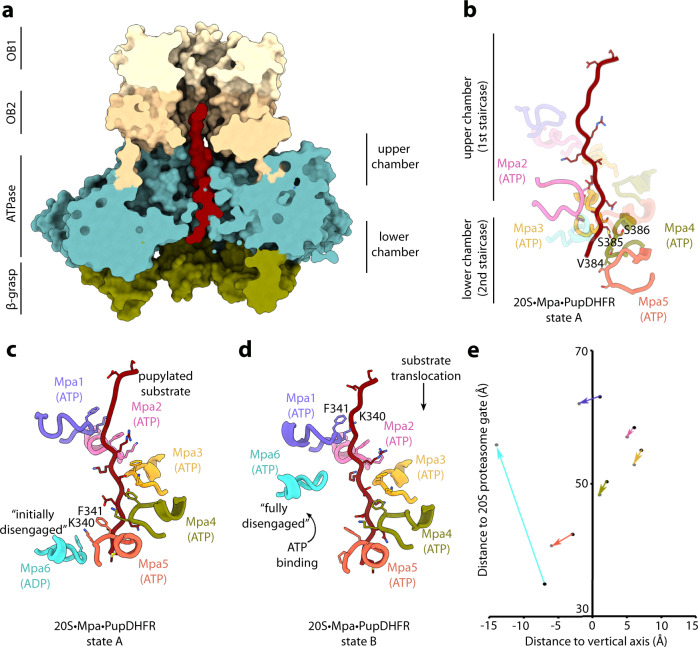
Spiral-staircase arrangement of Mpa protomer loops contacting the engaged substrate in the ATPase chamber. **a** The cross-section of the Mpa hexamer with the substrate peptide chain (red) visualizes the shape of the inner channel and the location of Pup inside the channel. Pore loop 1 (K340-E344) is located in the ATPase upper chamber and pore loop 2 (V384-S386) is located in the lower chamber**. b** Conformation of the pore loops in the ATPase lower chamber as resolved in state A is shown as cartoon with the contact residues (V384-S386) shown as sticks. The nucleotide state of the protomers is indicated. **c** Conformation of the pore loops in the ATPase chamber as resolved in state A is shown as cartoon with the contact residues (F341 and K340) shown as sticks. The nucleotide state of each protomer is indicated. **d** Conformation of the pore loops in the ATPase chamber as resolved in state B is shown as cartoon with the contact residues (F341 and K340) shown as sticks. The nucleotide state of each protomer is indicated. **e** Conformational changes in the pore loops as observed in state A and B are plotted based on the distance from the proteasome gate and distance from the vertical axis.

Our cryo-EM structure captures the Mpa–proteasome complex at the initial stage of substrate translocation, where the Mpa pore channel is occupied with the N-terminal segment of Pup (Fig. 5a). We could reliably model the first 15 amino acids of the Pup sequence into the electron density occupying the Mpa channel, indicating that the Mpa–20S CP complexes are uniformly occupied with this stretch of sequence (Fig. 5b). Both of the resolved states (state A and B) of the complex represent this early Pup-engagement intermediate (Supplementary Fig. 7a–c). A stacked pair of residues, the canonical aromatic pore loop residue F341 and the highly conserved adjacent lysine residue K340, forms a spiral-staircase that encircles the Pup N-terminal segment by engaging in unspecific interactions with its Cα backbone (Fig. 5c–e). Another channel loop about 16–17 Å further down (V384-S385-S386) forms a second spiral-staircase (Fig. 5b). It is resolved only in four of the six protomers, the other loops are flexible, as the Pup N-terminal segment has not yet reached far enough down at this state of engagement.

Interestingly, while in state A, all six of the canonical aromatic pore loops of the ATPase domain are in close proximity to the substrate backbone with one of them (protomer 6, cyan) initially disengaged (Fig. 5c), in state B only five protomers are in close proximity to the substrate backbone and engaged in interactions with it (Fig. 5d). The loop of the penultimate protomer (protomer 6, cyan), which was at the bottom of the staircase in state A, is retracted away from the substrate in state B and has shifted upward by 25 Å to a more distal position relative to the proteasome gate (Fig. 5c–e). Furthermore, in state B, the five pore loops contacting the substrate are shifted downwards by 2–3 Å compared to state A, which results in a translocation of the substrate towards the open gate of the proteasome by almost one amino acid.

The sequential cycles of substrate engagement and disengagement are tightly coupled with the nucleotide state of the ATP-hydrolyzing motor^41^. The achieved resolution of Mpa allowed the identification of the bound nucleotide present in each protomer of Mpa (Supplementary Fig. 8). However, we can only differentiate between bound trinucleotide (ATP or ATPγS) and dinucleotide (ADP), but for the trinucleotide we cannot distinguish between ATP and ATPγS. Since the reaction was quenched with ATPγS but initially incubated with ATP (see Methods), both would in principle be a possibility. Therefore, ATP or ATPγS will, for simplicity, be referred to as ATP.

The prominent position and strict conservation of lysine residue K340 in the translocation loop right adjacent to F341, suggests it plays a mechanistic role in the conformational cycle of loop movements and coupling to ATPase activity. In fact, K340 can form a salt-bridge with E344 from the neighboring protomer (Supplementary Fig. 9a), although this interaction is disrupted further down in the staircase, where the distance between the protomers increases. Interestingly, mutation of K340 to alanine abolishes the basal ATPase activity of Mpa on its own. This might point to a defect in the allosteric relay that stimulates the coordinated hydrolysis of ATP across the protomers in the Mpa ring. However, the ATPase activity is restored to 50% of wild-type level in the presence of both PupDHFR and proteasome (Supplementary Fig. 9b) and, in agreement with this, an alanine substitution variant, K340A, can still degrade PupDHFR model substrate in vitro, albeit at a mildly reduced rate (Supplementary Fig. 9c).

In state A, 5 of the 6 protomers of Mpa are in the ATP-bound state (Fig. 2a) with arginine fingers contributed by the neighboring subunit. The protomer with the pore loop at the bottom of the staircase (protomer 6, cyan) displays weak nucleotide density corresponding to the ADP nucleotide form. In this case, the arginine finger from the neighboring subunit is displaced and moved away from the bound nucleotide. There is also a prominent gap in the inter-subunit space between this protomer and the neighboring two Mpa protomers (Supplementary Fig. 8, left, bottom two panels). In state B in comparison, all protomers of Mpa are resolved with ATP bound, including protomer 6 (cyan) with its pore loop now in a disengaged and retracted conformation (Fig. 2b). Nevertheless, protomer 6 still exhibits less contacts to protomer 5, and the arginine finger that it contributes to protomer 5 does not contact the nucleotide, thereby creating a fissure in the ATPase ring between these protomers.

## Discussion

Actinobacteria acquired proteasomal genes and evolved a ubiquitin-like post-translational modification pathway to increase their survival under particularly challenging conditions, such as those encountered by Mtb inside host macrophages^43^. Centrally important to this pathway is the interaction of the AAA unfoldase Mpa with the 20S CP and the recruitment of protein substrates marked by pupylation for degradation by this molecular machine. Despite the significance of this complex as a drug target^44^, structural information on the fully assembled, active protease has been lacking. Our high-resolution cryo-EM structure of the substrate-engaged Mpa–proteasome complex reveals the dynamic character of the interaction between the AAA unfoldase ring and the proteasome core cylinder. It also delivers a molecular understanding of how Pup, the functional analog of ubiquitin in proteasomal targeting, engages with the Mpa pore to initiate substrate processing.

In eukaryotic cells, the 26S proteasome is responsible for the ATP-dependent turnover of protein substrates in the cytosol and nucleus. Together with the E1, E2, E3 enzyme cascade responsible for post-translational modification of target proteins with polyubiquitin chains, this recruitment and degradation pathway is referred to as the ubiquitin-proteasome system (UPS)^4,45,46^. The Pup-proteasome system (PPS) represents the bacterial functional analog of this degradation pathway^47,48^. The AAA unfoldase Mpa and the bacterial CP show homology to the eukaryotic regulatory ATPase subunits (Rpts) and the eukaryotic 20S, respectively. In contrast, bacterial ubiquitin-like targeting is an example of convergent evolution, and neither Pup and ubiquitin, nor the Pup ligase and the E1, E2, E3 cascade are homologous. Furthermore, the bacterial proteasomal unfoldase Mpa does not associate with additional non-ATPase subunits for substrate recruitment like it is the case for the eukaryotic 19S particle, where ubiquitin receptors Rpn1, Rpn10 and Rpn13 bind the polyubiquitin tag^49–51^. Instead, the intrinsically disordered Pup forms a long helix in the middle region of its primary structure (residues 22–50) that engages directly into an antiparallel, three-stranded coiled-coil with one of the N-terminal coiled-coil domains extending from the Mpa double-OB ring^19,31^. In addition to the poly-Ub tag, eukaryotic proteasome substrates additionally must feature an unstructured initiation region that can be threaded into the Rpt-ring pore for engagement by the translocation loops^52,53^, since Ub usually does not enter the ATPase motor, but is cleaved away by the deubiquitinase Rpn11 located close to the entrance pore^54^. In contrast, Pup as recruitment tag brings along the determinant for pore engagement in the form of its intrinsically disordered N-terminal region^8,32^.

Our structure trapped the Mpa–20S CP complex in the process of Pup-mediated substrate priming of the AAA motor. The N-terminal methionine of Pup (M1) is located in the lower Mpa chamber about 35 Å from the 20S gate. The Mpa β-grasp domains form a sort of collar around the 20S entrance gate, raising the AAA ring tier away from the α-ring surface by about 15 Å, whereas in contrast the Rpts in the 26S proteasome lie flat on the α-rings. Inside Mpa, the 15 resolved N-terminal Pup residues traverse a distance of about 50 Å from the second OB ring through the AAA ring-tier along the spiral-staircase. In the first OB ring, the density of substrate is not visible, but distance measurements show that Pup must span an additional 25 Å from the last resolved residue to the rim of the first OB ring (the pore entrance), corresponding to about seven residues in extended conformation. This would suggest that Pup residue 22 is located at or close to the entrance pore, and Pup is likely still engaged in the shared three-stranded coil with the Mpa coiled-coil domains. In the next step, ATPase-driven Pup translocation through the spiral-staircase of the Mpa AAA motor toward the 20S gate must overcome this interaction to allow translocation of unfolded Pup and DHFR into the proteasomal degradation chamber. This might explain why Pup is arrested in the Mpa channel in the same register across the particle population. Furthermore, the observation that substrate is stalled at this stage might indicate that this step presents a notable energy barrier and constitutes a rate-limiting step in the reaction cycle.

Recent developments in single-particle cryo-electron microscopy have enabled the determination of high-resolution structures of numerous substrate-engaged AAA+ machines including proteasome holoenzymes, and a common mechanism of substrate translocation has emerged^40,55–57^. In agreement with other substrate-engaged AAA+ ATPase structures, Mpa AAA motor pore loops adopt a spiral-staircase arrangement encircling the unfolded substrate, whereby the canonical aromatic F341 loop residue and the highly conserved K340 make the most prominent interactions with the polypeptide chain. It has been observed that a positively charged residue (K or R) frequently precedes the aromatic loop residue^41^. Across Actinobacteria the lysine in this position is strictly conserved, and a lysine is also found at this position in all but one of the Rpts of the eukaryotic proteasome. However, while the mutation of F341 is known to completely abolish substrate unfolding^8^, the K340A variant showed only a slightly lower rate of PupDHFR degradation. Interestingly, Mpa K340A in the absence of the 20S proteasome has an order of magnitude lower ATPase activity, pointing to a potential defect in the allosteric coordination of activity across subunits in the ring. Nevertheless, this defect can be overcome and activity restored to 50% of wild-type activity in the presence of both substrate and proteasome. It is possible that this residue is particularly important in the context of proteasome-independent roles played by Mpa, since the presence of substrate alone can only restore the activity to about 20% of wild-type activity. In *C. glutamicum*, for example, proteasomal subunits are absent, and Mpa was shown to be important for disassembly of ferritin during iron limitation^58^.

We resolved two distinct, sequential states of the Mpa complex with differences in spiral-staircase arrangement, nucleotide occupancy, and contacts between subunits and to the engaged substrate. Our observations agree with the increasing body of literature on substrate-bound structures of AAA+ motors pointing to a sequential, hand-over-hand mechanism^41^. In state A, the bottom protomer is in the ADP-bound state and displays a pronounced gap to the preceding subunit in the spiral, indicating it is in the process of disengaging from the substrate. Upon exchange of ADP for ATP, in state B, this protomer has moved to a higher position and is now disengaged from the substrate that has moved by about one residue length toward the proteasomal gate. State B can be seen as an intermediate state, and to complete the step, the disengaged subunit has to move into the topmost position, re-engaging the substrate at the top of the staircase, thereby resulting again in state A but with the protomers having advanced by one position in the ring. The two states are also consistent with previously observed states in the cryo-EM structures of substrate-engaged 26S proteasome^40,57^, Cdc48^56,59^, and ClpXP/AP^60–64^ structures. Ultimately, successive rounds of substrate engagements/disengagements would allow for substrate translocation into the CP chamber, where it is degraded.

Our structure demonstrates that the β-grasp domain in the C-terminal region of Mpa adopts a conformation that allows the GQYL motif to dock into the pockets of the proteasomal α-rings. The flexible anchoring of Mpa to the proteasome via the GQYL motif combined with the rail-like interactions between the β-hairpin loop and the H0 helices of the CP α-rings solves the problem of maintaining the necessary affinity between Mpa and the proteasome, while at the same time allowing the full range of conformational changes in Mpa that are associated with substrate unfolding and translocation into the proteasomal degradation chamber. The functional significance of the Mpa β-hairpin loop in providing charge-complementary contacts along the radial rail system generated by the H0 helices of the CP α-rings is further supported by the impairment of substrate degradation in the presence of the Mtb Mpa variant, where the native sequence of the loop was replaced with the one from *C. glutamicum*. In agreement with the role in proteasome interaction that we describe, residues 501–505 (ANGDK) constituting the β-hairpin are well conserved in those members of Actinobacteria that possess a 20S proteasome, but except for the central glycine important for the tight turn, the β-hairpin residues are not conserved in those species that lack proteasome core genes, like *C. glutamicum* (Supplementary Fig. 6).

The results presented here provide structural and mechanistic insights into the mycobacterial proteasome complex, revealing the molecular basis of the interaction between the hexameric AAA unfoldase Mpa and the proteasome core particle. Our study explains how the unfoldase and protease components can stably associate, while still allowing the AAA motor-mediated conformational changes required to unfold and translocate the substrate. Analogous mechanistic solutions may also exist in other chaperone-protease systems and may even be the reason for the evolutionarily conserved symmetry mismatch between the hexameric ATP-driven unfoldases and the heptameric proteolytic core particles, since the formation of symmetry-matched contacts between the two would likely interfere with the required conformational mobility of the AAA motor. Furthermore, our structure reveals how in mycobacteria pupylated substrates are engaged and translocated into the proteolytic chamber of the proteasome for degradation.

## Methods

### Generation of an unmarked *M. smegmatis mpa* deletion strain

Regions of roughly 1500 bp upstream and downstream of *mpa* were amplified from *M. smegmatis* MC^2^155 SMR5 genomic DNA by PCR (for primer sequences see Supplementary Table 2). Fragments were cloned into PCR-linearized p2NIL vector (p2NIL was a gift from Tanya Parish^65^; Addgene plasmid #20188) by isothermal DNA assembly. The resulting plasmid was digested with PacI and ligated with the PacI-excised marker cassette (hygR, lacZ, and sacB) from pGOAL19 (pGOAL19 was a gift from Tanya Parish^65^; Addgene plasmid #20190) to yield the *mpa* suicide plasmid. Suicide plasmid (1 μg) was UV-irradiated for 1 min before transformation into 300 μl *M. smegmatis* MC^2^155 SMR5 cells by electroporation (0.2 cm cuvette, 2.5 kV pulse, BioRad GenePulser). Electroporated cells were immediately recovered in 6.5 ml 7H9 shaking cultures for 6 h at 37 °C after which 150 μl culture were plated onto 7H10 agar supplemented with kanamycin (25 μg/ml) and hygromycin (50 μg/ml). Cells in the remaining culture volume were pelleted by centrifugation and plated as well. Colonies appeared after 3 days of incubation at 37 °C. Single-crossover colonies (SCOs) were identified by X-gal underlay (200 μl of 0.4% (w/v) X-gal in DMSO were spread underneath the agar and plates were incubated overnight at 37 °C). Four positive SCOs (blue color) were picked into 5 ml 7H9 supplemented with kanamycin (25 μg/ml) and hygromycin (50 μg/ml). SCO shaking cultures were grown at 37 °C for 2 days before diluting an aliquot to OD600 of 0.8 and preparing a 10-fold dilution series in 7H9. 100 μl of dilutions 1:1, 1:10, and 1:100 were plated on plain 7H10 agar and 7H10 agar supplemented with 2% (w/v) sucrose. Plates were incubated at 37°C for 3 days and subsequently analyzed by X-gal underlay. Positive double-crossover colonies (DCOs; white color) were screened for *mpa* deletion by colony PCR, streaked on plain 7H10 agar and 7H10 supplemented with kanamycin (25 μg/ml) and grown at 37 °C. Kanamycin-sensitive DCOs identified as bearing the *mpa* deletion in the colony PCR screen were further examined by amplification and sequencing of the *mpa* genomic locus. Of the remaining candidates, one DCO was selected, grown in 7H9 liquid culture followed by preparation of glycerol stocks which were frozen in liquid nitrogen and stored at −80°C until use. The absence of the Mpa protein in the cellular lysate of the newly generated knockout strain was further confirmed by immunoblotting.

### Bacterial strains and culture conditions

Strains used were *M. smegmatis* MC^2^155 SMR5 and MC^2^155 SMR5 Δ*mpa*. Bacteria were routinely grown in Middlebrook 7H9 (supplemented with 0.2% v/v glycerol and 0.05% w/v Tween-80) or on Middlebrook 7H10 agar plates. If applicable, hygromycin B (Carl Roth) was added to a final concentration of 50 μg/mL. Liquid cultures were routinely grown at 37 °C in a shaking incubator and agar plates were incubated at 37 °C for growth.

### Alignment

Protein sequence alignment was carried out using the Clustal X2.1 multiple sequence alignment software^66^. ESPript 3.0 was used to generate the graphical representation of the alignment^67^.

### Cloning, expression and purification of proteins

The sequence of Mtb *mpa* encoding the untagged, wild-type protein was previously cloned into pET20 (Novagen) via NdeI and BamHI restriction sites to generate the pET20-Mpa overexpression plasmid^8^. The plasmids coding for Mpa variants were obtained from pET20-Mpa by site-directed mutagenesis.

The plasmid for overexpression of PupDHFR was constructed by Gibson assembly (NEB) from the previously cloned pET20 plasmid coding for Mtb Pup with an N-terminal His_10_-Thioredoxin fusion (His_10_-Thioredoxin-MtbPupE) and the PCR fragment containing the *E. coli* dihydrofolate reductase gene. The sequences of primers used in this study are provided in Supplementary Table 2.

Sequences encoding Mtb Δ7PrcA and Mtb Δ57PrcB (α-subunit is N-terminally truncated by seven residues, β-subunit propeptide sequence is removed) were cloned into a single pET-Duet-1 (Novagen) overexpression plasmid with Δ57PrcB forming a C-terminal fusion with the Strep-tag sequence to allow for simultaneous expression of both proteins and isolation of the assembled 20S CP by affinity purification. The plasmid coding for proteasome variant Δ7CP_H0-3RA was generated from pET-Duet-1-Δ7PrcA-Δ57PrcB by site-directed mutagenesis.

Mpa variants were expressed in *E. coli* Rosetta (DE3) cells at 30 °C with IPTG induction (0.1 mM, 4 h). Cells were harvested (F9S, 7000 rpm (9180 g), 10 min, 4 °C), cell pellets were frozen with liquid N_2_ and stored at −20 °C until purification. Cell pellets were resuspended in buffer FFQ-A (50 mM HEPES/KOH pH 7.5, 50 mM NaCl, 2 mM EDTA, 10% glycerol, 1 mM DTT) supplemented with 1X PMSF (1 mM) and 1X cOmplete protease inhibitor (Roche), and the cells were lysed by high pressure shear force using a Microfluidizer M110-L device (Microfluidics; 5 passes, 11,000 psi chamber pressure). After removal of cell debris (SS34, 20,000 rpm (47,810 g), 4 °C, 1 h), cleared lysate was separated by anion-exchange chromatography (Fast Flow Q 65 mL; buffer FFQ-A: see above; buffer FFQ-B: same as FFQ-A but 1 M NaCl; gradient 8–60% of FFQ-B over 8 CV) and Mpa-containing fractions were pooled, precipitated with 60% (NH_4_)_2_SO_4_, redissolved, dialyzed and further purified by cation-exchange (Source 30 S 20 mL; buffer 30S-A: 25 mM MES/KOH pH 6.0, 10% glycerol, 1 mM DTT, 2 mM EDTA; buffer 30S-B: same as 30S-A but 1 M NaCl; gradient 0–30% of 30S-B over 15 CV) and size-exclusion chromatography (Superose 6; 50 mM HEPES/KOH pH 7.5, 150 mM NaCl, 10% (v/v) glycerol, 1 mM EDTA, 1 mM DTT). Mpa variants were stored at −20 °C in 50 mM HEPES/KOH pH 7.5, 150 mM NaCl, 10% (v/v) glycerol, 1 mM EDTA, 1 mM DTT.

Pup(E)DHFR was expressed in *E. coli* Tuner (DE3) cells at 37 °C with IPTG induction (0.2 mM) as His_10_-Thioredoxin-TEV-Pup(E)-DHFR fusion protein and purified by Ni^2+^-affinity chromatography. After cleavage of the fusion protein with TEV-protease, His_10_-Thioredoxin and TEV-protease were removed by Ni^2+^-affinity chromatography. Pup(E)DHFR was stored in 50 mM Tris base/HCl pH 7.5, 150 mM NaCl, 1 mM EDTA.

Wild type and H0-3RA variants of open-gate 20S proteasome were expressed in *E. coli* Rosetta (DE3) cells at 25 °C overnight with IPTG induction (0.1 mM) and purified by affinity chromatography with StrepTactinXT resin (IBA Lifesciences) followed by size-exclusion chromatography (Superose 6). The purified open-gate proteasomes were stored at 4 °C in 50 mM HEPES/KOH pH 7.5 4 °C, 150 mM NaCl, 1 mM EDTA, 10% glycerol.

All protein concentrations were determined by absorbance spectroscopy at 280 nm. Mpa concentrations are provided in terms of hexamers. Proteasome concentrations are given in terms of assembled core particle.

### Proteasomal degradation assays

Open-gate proteasome variant (0.2 μM), Mpa or Mpa variant (0.2 μM) and PupDHFR (2 μM), were mixed in buffer R (50 mM HEPES/KOH pH 7.5 4 °C, 150 mM NaCl, 5% glycerol, 10 mM KCl, 20 mM MgCl_2_, 1 mM DTT). The reaction was started by the addition of ATP (10 mM) in a total volume of 150 μL and was carried out at 37 °C. Aliquots were collected at the indicated time points and analyzed by SDS-PAGE (sodium dodecyl sulfate–polyacrylamide gel electrophoresis). Experiments were repeated independently three times yielding similar results. Source Data (uncropped gels) are provided in the Source Data file.

### Complementation studies of *M. smegmatismpa* knockout strain and time-resolved RecA degradation assay

*M. smegmatis* Δ*mpa* strain was complemented with either wild type or mutant Mpa under control of the native *mpa* promotor using modified pMyNT vectors containing the L5 integrase gene and grown in 100 mL shaking cultures at 37 °C in 7H9 medium in the presence of hygromycin B (50 μg/mL). Cells were subjected to DNA damage stress by addition of mitomycin C (MMC, 80 ng/mL) at an OD600 of 1.0 to 1.2. After 30 min incubation with MMC, cells were harvested by centrifugation (3500 *g*, 10 min, 25 °C), washed once in 100 mL fresh 7H9 medium, resuspended in 100 mL fresh 7H9 medium, and transferred to a new, sterile flask to ensure complete removal of MMC. Cultures were incubated for another 48 hr, 5 mL aliquots were withdrawn at the indicated time points and cells were collected by centrifugation (3500 *g*, 5 min, 4 °C). Pellets were immediately flash-frozen in liquid N_2_ and stored at −20 °C for later analysis. Pellets were then resuspended in 800 μl lysis buffer (50 mM HEPES/KOH pH 7.5, 150 mM NaCl, 1 mM EDTA, 1 mM PMSF, 1X cOmplete EDTA-free protease inhibitor (Roche) and lysed by bead beating in 2 ml screw cap tubes containing 500–700 mg 0.15 mm zirconium oxide beads using an MP Biomedicals FastPrep-24 bead beater (3 × 30 s, 6 m/s, 1 min pause on ice in between). After removing insoluble material by centrifugation (16,000 g, 10 min, 4 °C), protein content of the cleared lysate was determined by Bradford assay. 5 μg of total protein were separated on a Mini-PROTEAN stain-free 4–20% gradient polyacrylamide gel (BioRad) and transferred onto PVDF membrane Immobilon-P (Merck) using a BioRad Trans-Blot SD semidry transfer cell (20 V, maximum of 10 mA/cm^2^, 30 min). Membranes were blocked in PBS containing 0.05% (v/v) Tween-20 and 5% (w/v) nonfat dried milk for 1 h at room temperature. Immunoblotting for RecA and RpoB was carried out using commercially available anti-RecA antibody (1:2000, MBL International, clone ARM414) raised in mouse or commercially available anti-RpoB antibody (1:5000, BioLegend, clone 8RB13) raised in mouse^68^. The blocked membrane was incubated with primary antibodies in blocking buffer with 0.5% (w/v) nonfat dried milk for 1 h at room temperature. Detection was achieved by incubation with horseradish peroxidase-conjugated anti-mouse IgG antibody (1:5,000, Promega #W4021) in blocking buffer with 0.5% (w/v) nonfat dried milk for 1 h at room temperature and using ECL substrate (BioRad Clarity Western Substrate). Three complete, individual experiments were carried out, each starting from a single colony of freshly transformed and plated *M. smegmatis* Δ*mpa* cells, one representative is shown in Fig. 4b. Source Data (uncropped and unprocessed blots) are provided in the Source Data file.

### ATPase activity of Mpa variants

ATPase activity of wild-type Mpa and Mpa variants was measured at 23 °C in buffer R with a continuous spectrophotometric assay coupled to inorganic phosphate production using 7-methylinosine and the enzyme purine nucleoside phosphorylase^69^. The calibration curve was recorded in the presence of PNPase (1.4 U), 7-methylinosine (1 mM) and 0–600 μM K_2_HPO_4_. The absorbance was monitored at 291 nm until the plateau was reached and the A_291_ value at the plateau was recorded for each phosphate concentration and used to construct the calibration curve. The reaction contained Mpa variant (0.1 μM), PNPase (1.4 U), 7-methylinosine (1 mM) and was started by addition of ATP (10 mM). The absorbance was monitored at 291 nm, the linear part of the curve was used to derive the ATPase activity. The ATPase activity of the Mpa K340A variant was additionally measured in the presence of open-gate proteasome (0.1 μM), PupDHFR (1 μM) or both open-gate proteasome and PupDHFR. The plots showing the measured ATPase activities were generated with GraphPad Prism 7.04.

### EM specimen preparation and imaging

Open gate proteasome (3 μM), Mpa (3 μM), ATP (5 mM), 2x reaction buffer R and ddH_2_O were mixed in a total volume of 400 μL and incubated at 37 °C for 2 min. The sample was then loaded onto a Superose 6 10-300GL gel filtration column pre-equilibrated in buffer R without glycerol at 4 °C. The same buffer was used as running buffer. The elution fraction containing the Mpa–20S CP complex was concentrated using a 0.5 mL Amicon spin filter (100 kDa MWCO) to approximately 2 mg/mL.

Mpa–proteasome sample (1.09 mg/mL), PupDHFR (1.06 mg/mL) and ATP (1.76 mM) were mixed together and incubated for 60 s on ice. ATPγS (5.4 mM) and NP-40 (0.003%) were added and the resulting sample was immediately applied on the grid.

Sample of the assembled complex (5 μL) was applied to holey carbon R2/1 Quantifoil grids that were glow discharged with a Pelco EasyGlow system for 30 s prior to use. Grids were immediately blotted with filter paper and then plunged into liquid ethane/propane mix cooled to liquid nitrogen temperature using a ThermoFisher Vitrobot at 4 °C with 95% relative humidity. Data collection was performed on a Titan Krios electron microscope (ThermoFisher) operated at 300 kV, using the EPU software (ThermoFisher) for automated data acquisition in counting mode using the Gatan K3 direct electron detector with an energy filter slit width of 20 eV. Data were collected at a defocus of −1.2 to −2.5 μm range with step size of 0.1 μm and at a nominal magnification of ×105,000, which resulted in a calibrated pixel size of 0.84 Å/pixel (0.42 Å/pixel in super-resolution mode). Illumination conditions were adjusted to an exposure rate of 32 e-/pixel/second. Micrographs were recorded as movie stacks with an electron dose of ~50 electrons/Å^2^ fractionated into a total of 40 frames. Drift and bright gain reference corrections in addition to dose-weighting were performed with MotionCor2^70^.

### Cryo-EM data processing and refinement

Contrast transfer function (CTF) was calculated using GCTF^71^ and the power spectra of the micrographs were then carefully inspected for drift, and images with signal extending to less than 5 Å were discarded from the two datasets. Particle images (860,718) were selected using the Laplacian Gaussian Blob as a reference as implemented in RELION3^72^ from a total of 12,673 micrographs.

Initial processing of the images was done with CryoSPARC^73^. First, two-dimensional (2D) image classification was performed on binned images. An ab-initio refinement was then performed on selected particles (385,394) from 2D class-averages that resolved features of the proteasome, asking for five initial starting models. The initial model that depicted a capped proteasome was then used as a reference for a heterogeneous 3D refinement in CryoSPARC asking for six 3D classes. Particles (222,719) in the 3D class which displayed the capped 20S CP were selected. Other classes showing empty 20S CP or weak Mpa densities were discarded. To improve the local resolution of Mpa, the signal of the 20S CP was subtracted from each image, and aligned Mpa particles were subjected to 3D classification in RELION3. The particles in the two classes with the best-resolved features of Mpa were refined using signal-subtracted images in RELION3. To generate the 20S CP subtracted images, a soft mask was generated based on the Mpa density, which allowed the density of the 20S CP to be subtracted from individual particles and then the subtracted images to be re-centered on the center of mass of Mpa in RELION3. The center of mass was determined on masked Mpa by using the “shift_com” option using the relion_image_handler command^72^ resulting in shift values *x* = −0.6, *y* = −2.6, and *z* = −78 relative to the center of the box. Re-centered and subtracted images were then refined using a masked Mpa obtained from the 3D classification as an initial reference. The resulting maps of Mpa state A and state B, refined to 3.8 and 3.9 Å resolution, were shifted back to the original position before re-centering. Validation of the relative positions was carried out by rigid body fitting of the focused Mpa maps into the globally refined map of the Mpa–20S CP, which only resulted in minor shifts in the individual focused maps without any rotations or tilts. Composite maps of Mpa and 20S CP were then generated by using the “add” option. To obtain a reconstruction of the 20S CP, a tight mask was applied on the 20S CP and particles were 3D refined in RELION3, which resulted in a final reconstruction of 2.8 Å resolution. All final 3D refinements and map sharpening were performed in RELION with a binned pixel size of 1.22 Å/pixel. Local resolution and gold standard FSC plots using FSC = 0.143 as a criterion were calculated in RELION3 (Supplementary Fig. 2).

### Model building in the cryo-EM maps

For model building into the cryo-EM map, the coordinates of mycobacterial 20S CP (PDB ID:5LZP [10.2210/pdb5LZP/pdb]^74^) were docked as rigid body into the cryo-EM map using USCF CHIMERA, and adjusted based on their side chain/secondary structure densities. The GQYL-motif residues were built into their corresponding EM densities and adjusted in COOT^75^. For model building into the cryo-EM maps of Mpa states A and B, coordinates of one Mpa protomer (PDB ID: 5KWA [10.2210/pdb5KWA/pdb]^28^) were used as a starting model and then divided into the OB ring, ATPase, helical, and β-grasp domains and docked as rigid bodies into each of the 6 protomers in states A and B. The coordinates were then adjusted based on the visible secondary structure elements and side-chain EM densities. The ATPase pore loops, which contact the substrate, were built de novo into the corresponding cryo-EM densities of both states A and B. The bound ATP or ADP nucleotides were placed based on their visible densities in the cryo-EM maps. The EM-density corresponding to the N-terminus of Pup was built de novo based on the visible side-chain density in COOT. To generate a full model for the 20S CP–Mpa complex, a composite EM map was generated by adding the 20S CP and Mpa EM maps from the focused 3D refinements in RELION. The models of Mpa state A or B and of the 20S CP were then docked as rigid bodies. For model refinements, all resulting models were then refined into the corresponding EM densities and subjected to eight cycles of real space refinements using phenix.real_space_refine in PHENIX^76^, during which protein secondary structure, Ramachandran and side-chain rotamer restraints were applied. The fit of the EM map was validated using the real space correlation coefficients (CCmask) between the model versus the map Fourier Shell Correlation (FSC) at FSC = 0.5 as a cut-off criterion, which resulted in similar resolution as the half-set map FSC using FSC = 0.143 as a cut-off criterion. Images were prepared in either ChimeraX^77^ or PyMOL^78^.

### Reporting summary

Further information on research design is available in the Nature Research Reporting Summary linked to this article.

## Supplementary information

Supplementary Information

Reporting Summary

## Data Availability

Protein structure data and the coordinates generated in this study have been deposited in the Protein Data Bank with PDB accession codes 7PX9 (Substrate-engaged Mpa in state A-focused 3D refinement), 7PXA (open-gate 20S CP—global 3D refinement), 7PXB (Substrate-engaged Mpa in state B—focused 3D refinement), 7PXC (Substrate-engaged Mpa in state A in complex with open-gate 20S CP—composite map) and 7PXD (Substrate-engaged Mpa in state B in complex with open-gate 20S CP—composite map). Cryo-EM maps have been deposited with the Electron Microscopy Data Bank as EMD-13694 (Substrate-engaged Mpa in state A-focused 3D refinement), EMD-13695 (open-gate 20S CP—global 3D refinement), EMD-13696 (Substrate-engaged Mpa in state B—focused 3D refinement), EMD-13697 (Substrate-engaged Mpa in state A in complex with open-gate 20S CP—composite map) and EMD-13698 (Substrate-engaged Mpa in state B in complex with open-gate 20S CP—composite map). Source data are provided with this paper.

## Source data

Source Data
