# Supplementary material for: Structural basis of prokaryotic ubiquitin-like protein engagement and translocation by the mycobacterial Mpa-proteasome complex
Source: Nat Commun. 2022 Jan 12;13:276. doi: 10.1038/s41467-021-27787-3 (PMC8755798; doi:10.1038/s41467-021-27787-3)
Supplement: Supplementary file 1 — Supplementary Information [file 41467_2021_27787_MOESM1_ESM.pdf]

Structural basis of prokaryotic ubiquitin-like protein engagement and translocation by the mycobacterial Mpa-proteasome complex

Mikhail Kavalchuk[1,#], Ahmad Jomaa[1,#,*], Andreas U. Müller[1] & Eilika Weber-Ban[1,*]

[1]ETH Zurich, Institute of Molecular Biology & Biophysics, CH-8093 Zurich, Switzerland

* Address correspondence to E.W.-B. (eilika@mol.biol.ethz.ch) or A.J. (ahmad.jomaa@mol.biol.ethz.ch)

[#] These authors contributed equally.

– Supplementary Information –

SUPPLEMENTARY FIGURES
Supplementary Figure 1

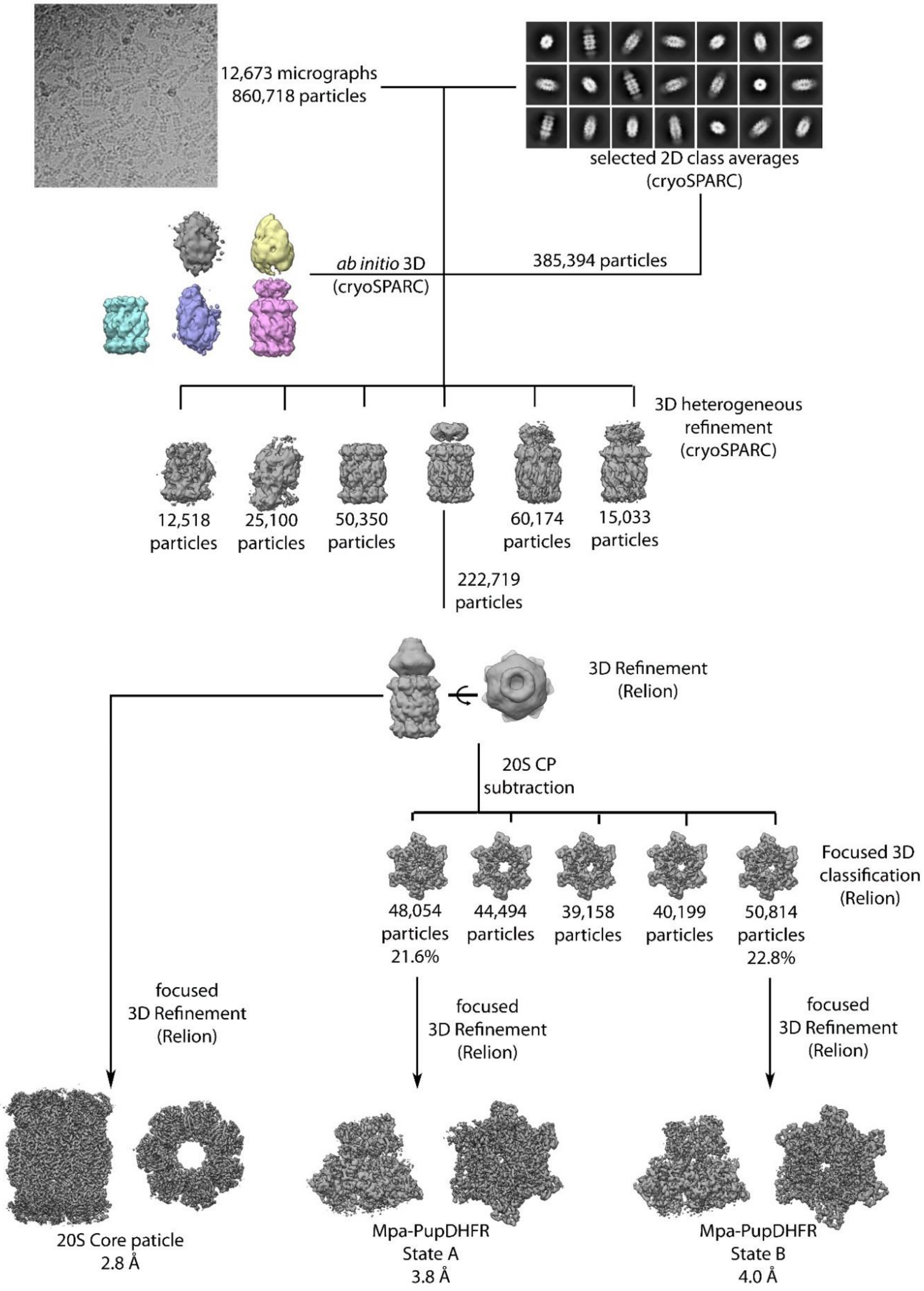

Supplementary Figure 1. Schematic representation of the cryo-EM single-particle data processing. Initial 2D and 3D classifications of particle images, and *ab initio* model determination were performed in CryoSPARC on binned images with a pixel size corresponding to 3.36 Å. Final 3D refinements on focused regions of the complex (Mpa or CP) were done in RELION on images with a pixel size corresponding to 1.22 Å.

Supplementary Figure 2

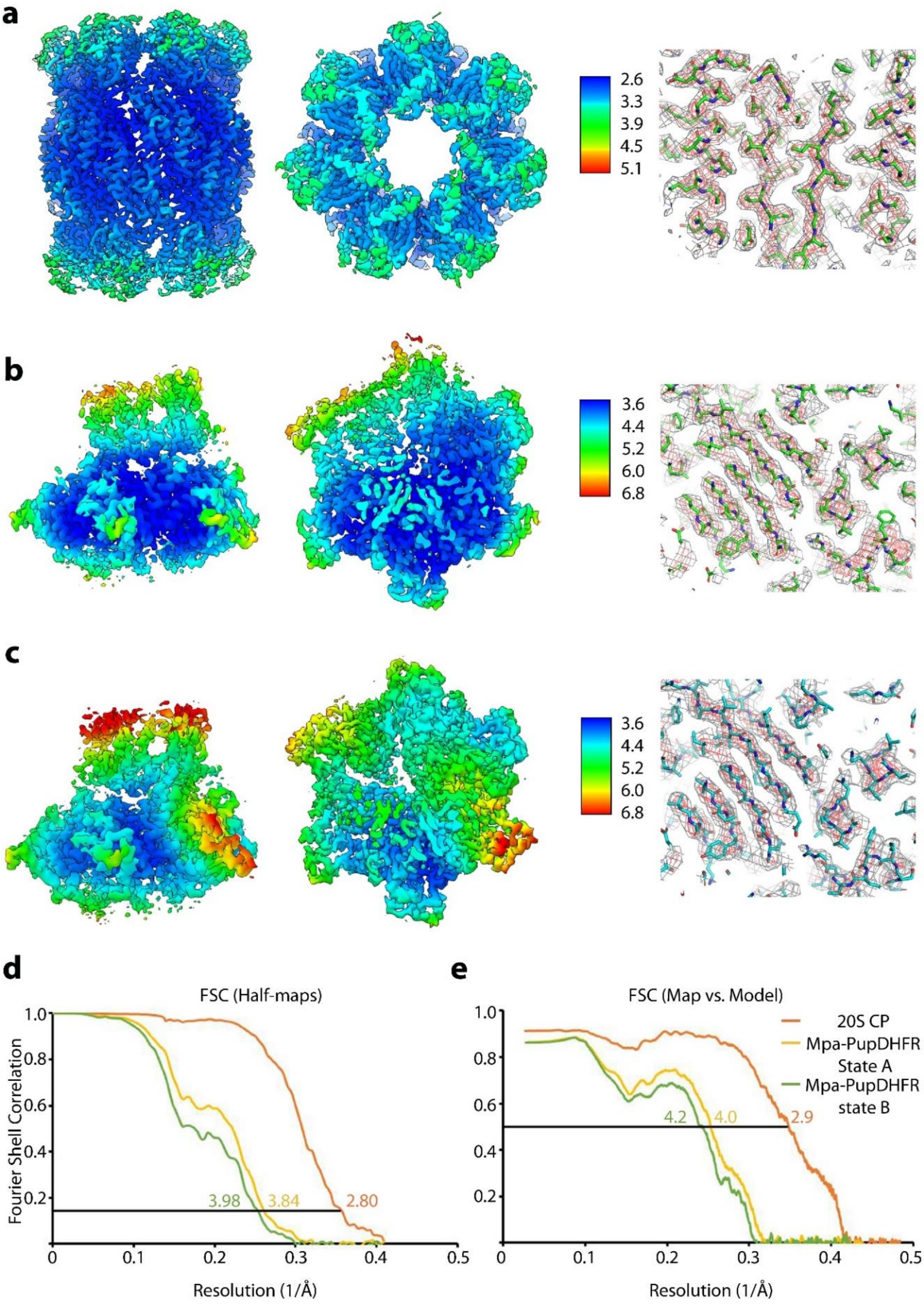

Supplementary Figure 2. Local resolution and FSC plots of the maps. a-c, Local resolution estimates are calculated in RELION and shown for the reconstructions of the 20S CP, Mpa states A and B. Left panels depict close-ups of distinct regions of the reconstruction with underlying atomic models. **d**, Fourier Shell Correlation (FSC) plots for the three reconstructions calculated between the half maps calculated in RELION. The cut-off at 0.143 is indicated by a black line. **e**, FSC plots for the model versus map are calculated in PHENIX. The cut-off at 0.5 is indicated by a black line.

Supplementary Figure 3

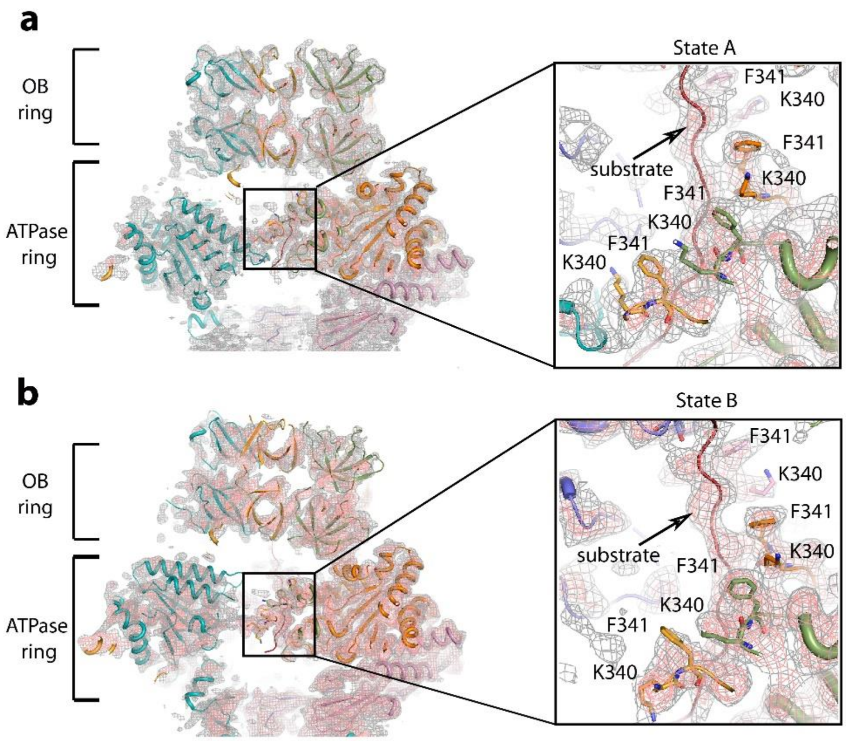

Supplementary Figure 3. Spiral staircase conformation of the pore loops in states A and B. a-b, Cross-section of Mpa depicting the ATPase chamber with the pore loops in an engaged conformation with the pupylated substrate (maroon; indicated with an arrow) in a spiral staircase conformation. Residues K340 and F341 of the pore loops are indicated for each protomer in state A (panel a) and state B (panel b). EM-densities are shown as mesh with underlying atomic coordinates shown as cartoon and sticks.

Supplementary Figure 4

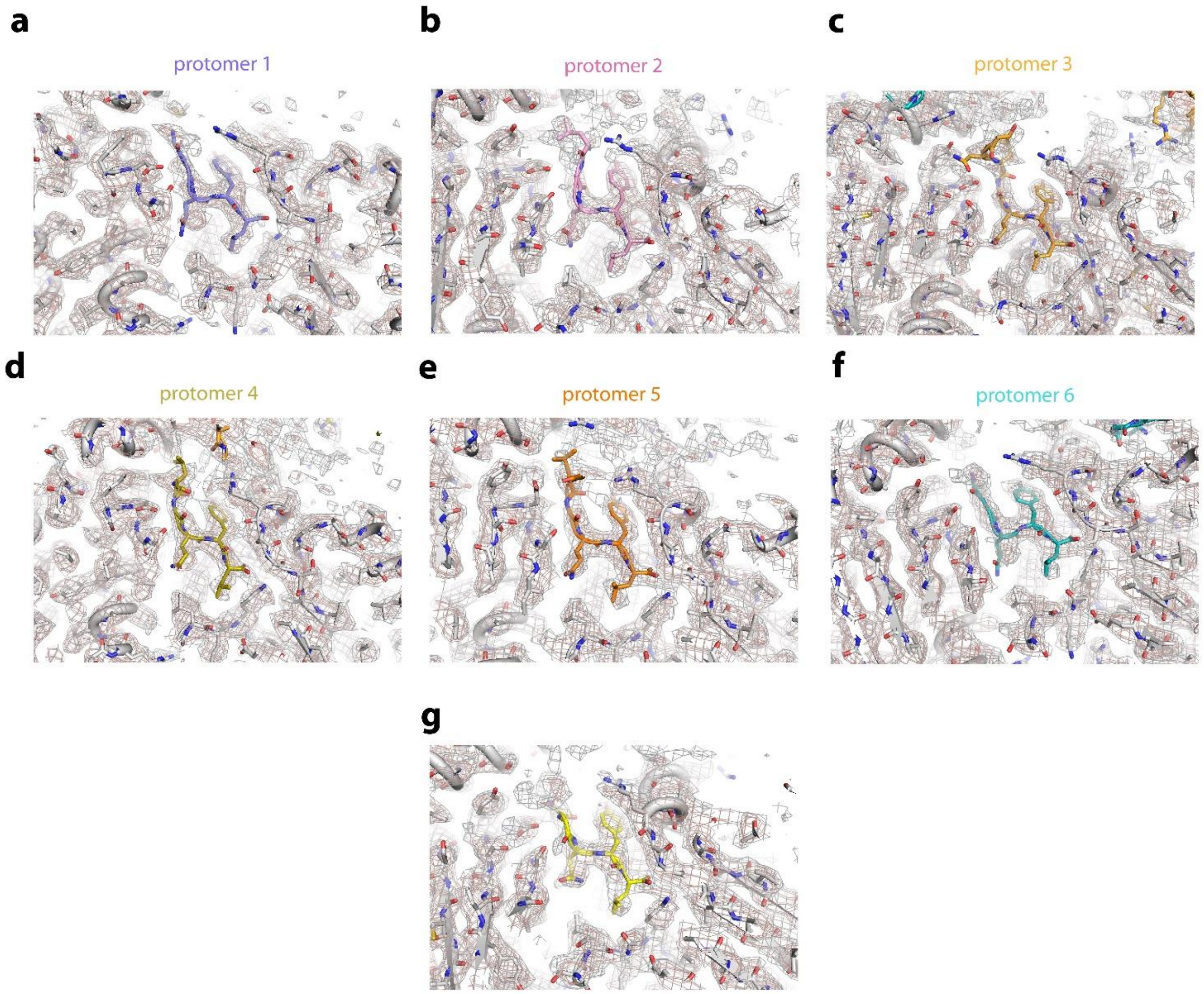

Supplementary Figure 4. Close-ups of the GQYL motif bound to the proteasomal α-ring pockets. a-g, Close-ups of the EM-density shown as mesh with underlying atomic coordinates shown as sticks. Each density attributed to a protomer of Mpa is indicated and color-coded as in Fig. 1. One additional tail (panel g; bright yellow) could not be attributed to an Mpa protomer and was left as an unassigned chain. Coordinates of the proteasome are colored light grey.

Supplementary Figure 5

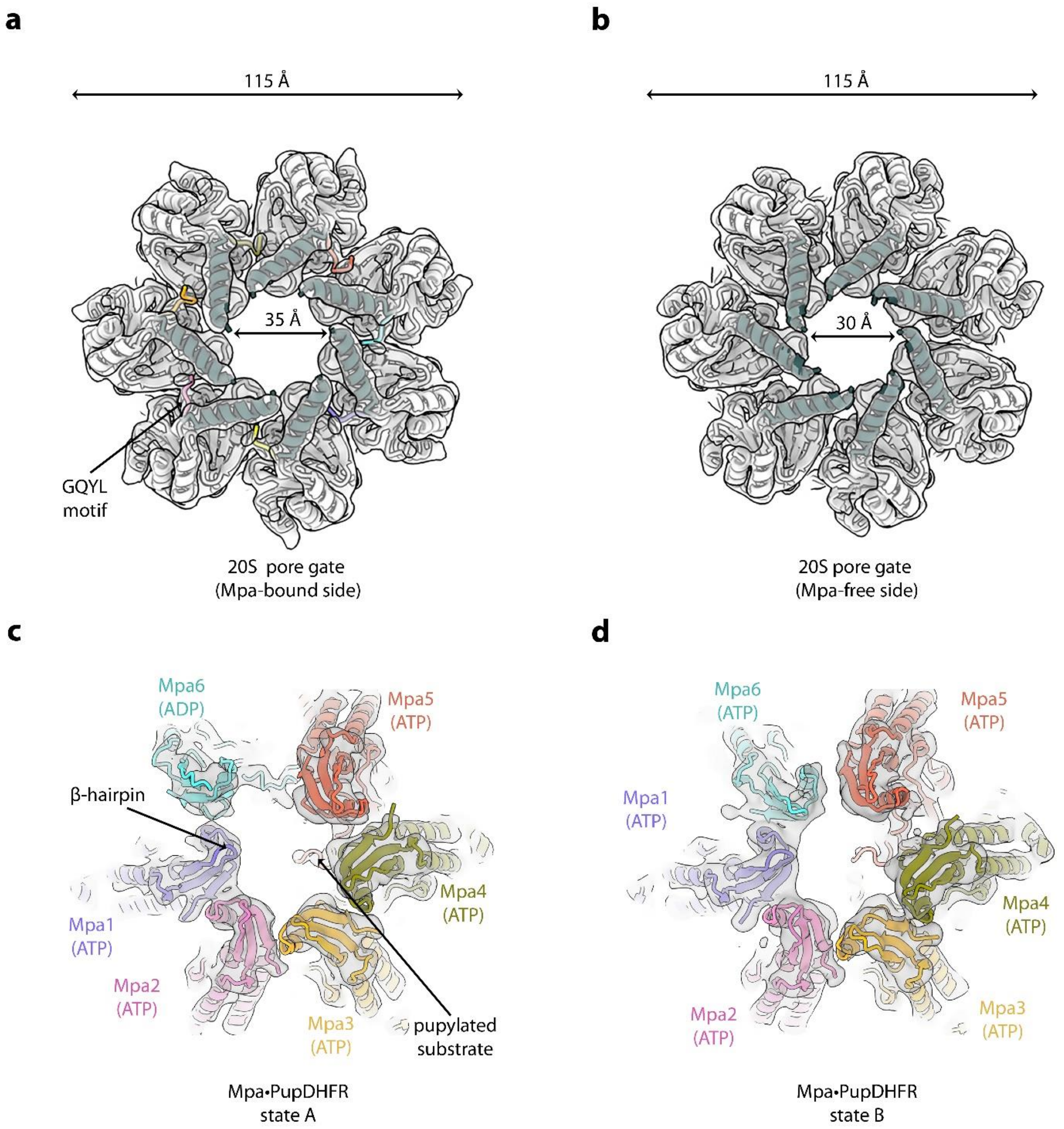

Supplementary Figure 5. Top view of proteasomal α-rings and bottom view of the Mpa ring. a-b, Top views of the proteasomal α-rings in the 20S CP from Mpa-bound (a) and Mpa-free (b) sides showing pore diameter measured at the proteasomal gate. The position of one of the GQYL motifs (colored according to Mpa protomer color code) is indicated with an

arrow. **c-d**, Bottom view of the Mpa ring in state A (c) and state B (d) with cryo-EM maps shown as transparent surface and low-pass filtered to 6 Å resolution with underlying atomic coordinates shown as cartoon. The β-hairpin and the engaged substrate are indicated with an arrow.

Supplementary Figure 6

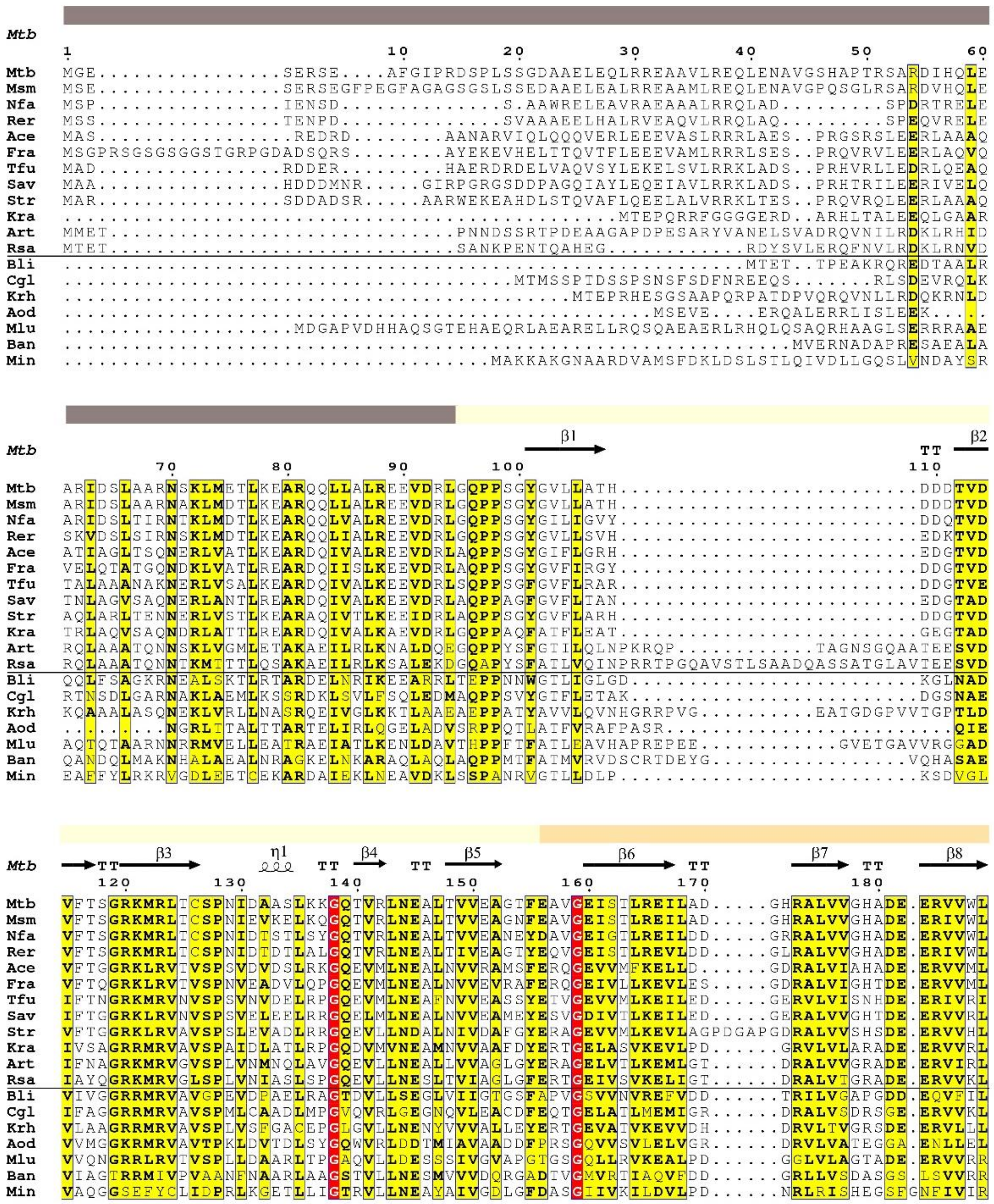

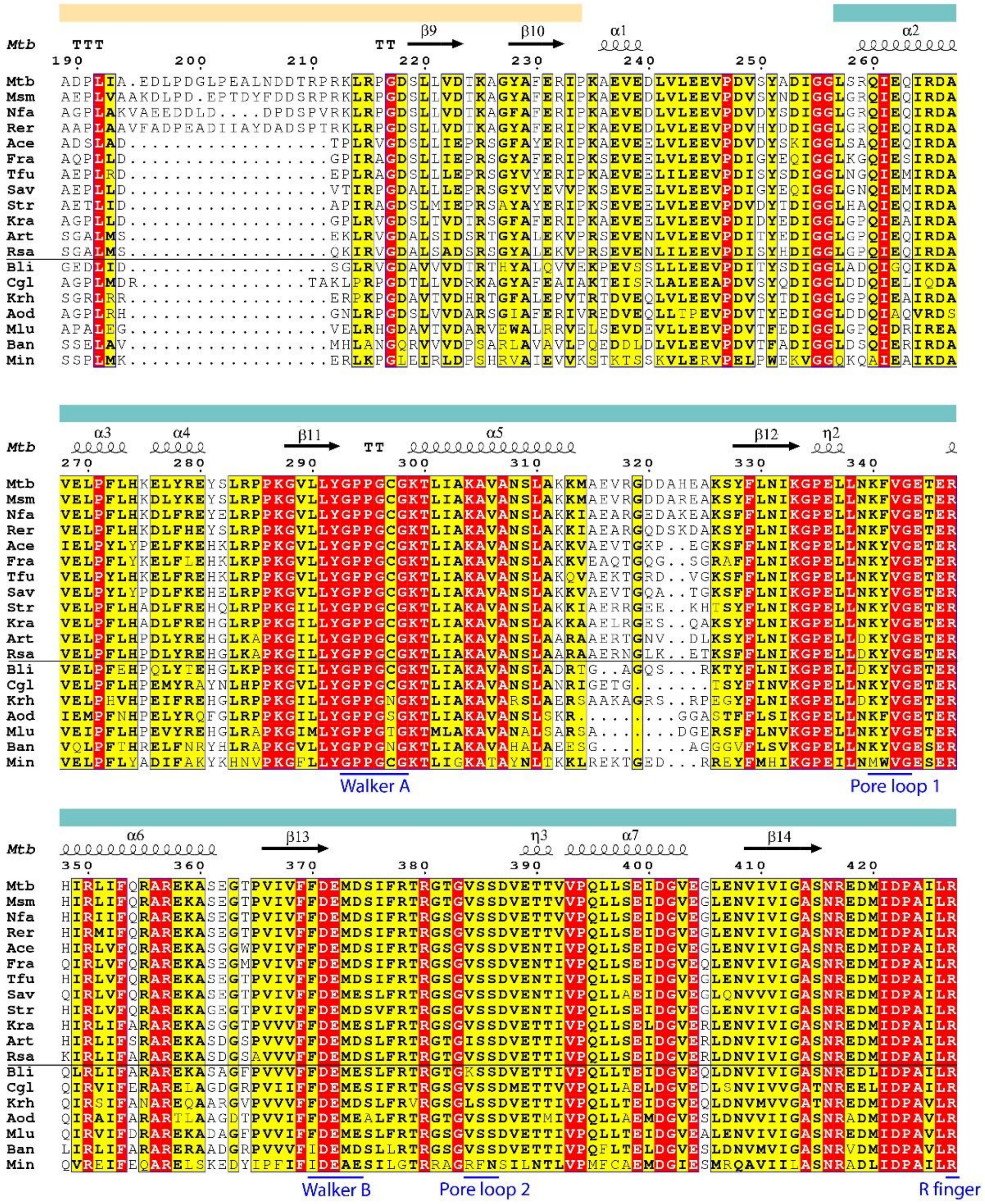
Mtb
Msm
Nfa
Rer
Ace
Fra
Tfu
Sav
Str
Kra
Art
Rsa
Bli
Cgl
Krh
Aod
Mlu
Ban
Min
Walker A
Pore loop 1
Walker B
Pore loop 2
R finger

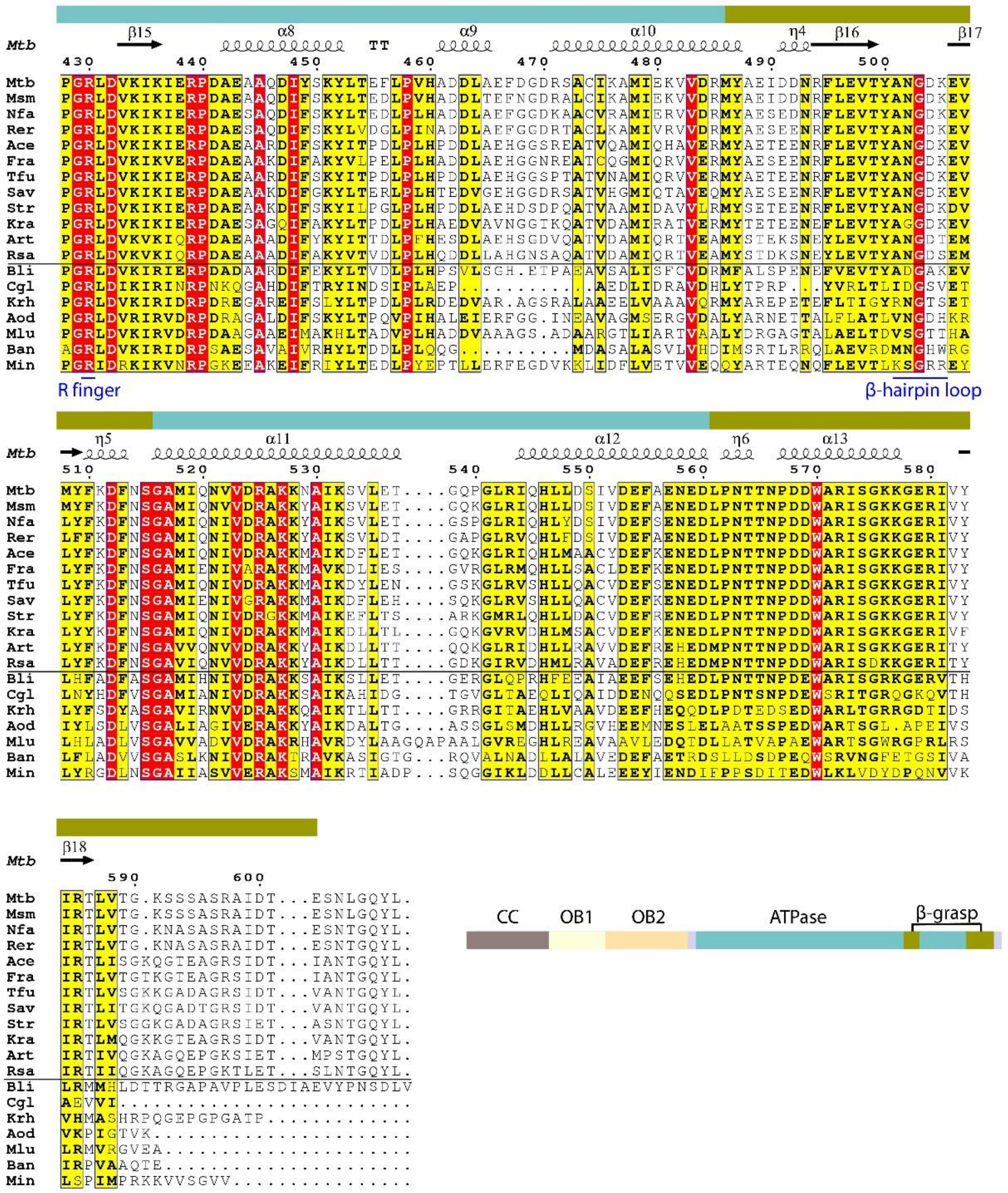
R finger
β-hairpin loop
CC
OB1
OB2
ATPase
β-grasp

Supplementary Figure 6. Multiple sequence alignment of Mpa (ARC) from various actinobacterial species. *Mycobacterium tuberculosis* H37Rv (Mtb), *Mycobacterium smegmatis* MC^2 155 (Msm), *Nocardia farcinica* IFM 10152 (Nfa), *Rhodococcus erythropolis* PR4 (Rer), *Acidothermus cellulolyticus* 11B (Ace), *Frankia* sp. EAN1pec (Fra), *Thermobifida fusca* YX (Tfu), *Streptomyces avermitilis* MA-4680 (Sav), *Salinispora tropica* CNB-440 (Str), *Kineococcus radiotolerans* SRS30216 (Kra), *Arthrobacter* sp. FB24 (Art), *Renibacterium salmoninarum* ATCC 33209 (Rsa), *Brevibacterium linens* BL2 (Bli), *Corynebacterium glutamicum* ATCC 13032 (Cgl), *Kocuria rhizophila* DC2201 (Krh), *Actinomyces odontolyticus* ATCC 17982 (Aod), *Micrococcus luteus* NCTC 2665 (Mlu), *Bifidobacterium angulatum* DSM 20098 (Ban), *Methylacidiphilum infernorum* V4 (Min). The black horizontal line separates the species possessing both proteasome and Mpa/ARC (above the line) from those possessing only Mpa/ARC but not the proteasome (below the line). The colors of the schematic representation of Mpa are in agreement with Fig. 5 D; CC – coiled-coil domain, OB1 and OB2 – oligonucleotide-binding domains 1 and 2.

Supplementary Figure 7

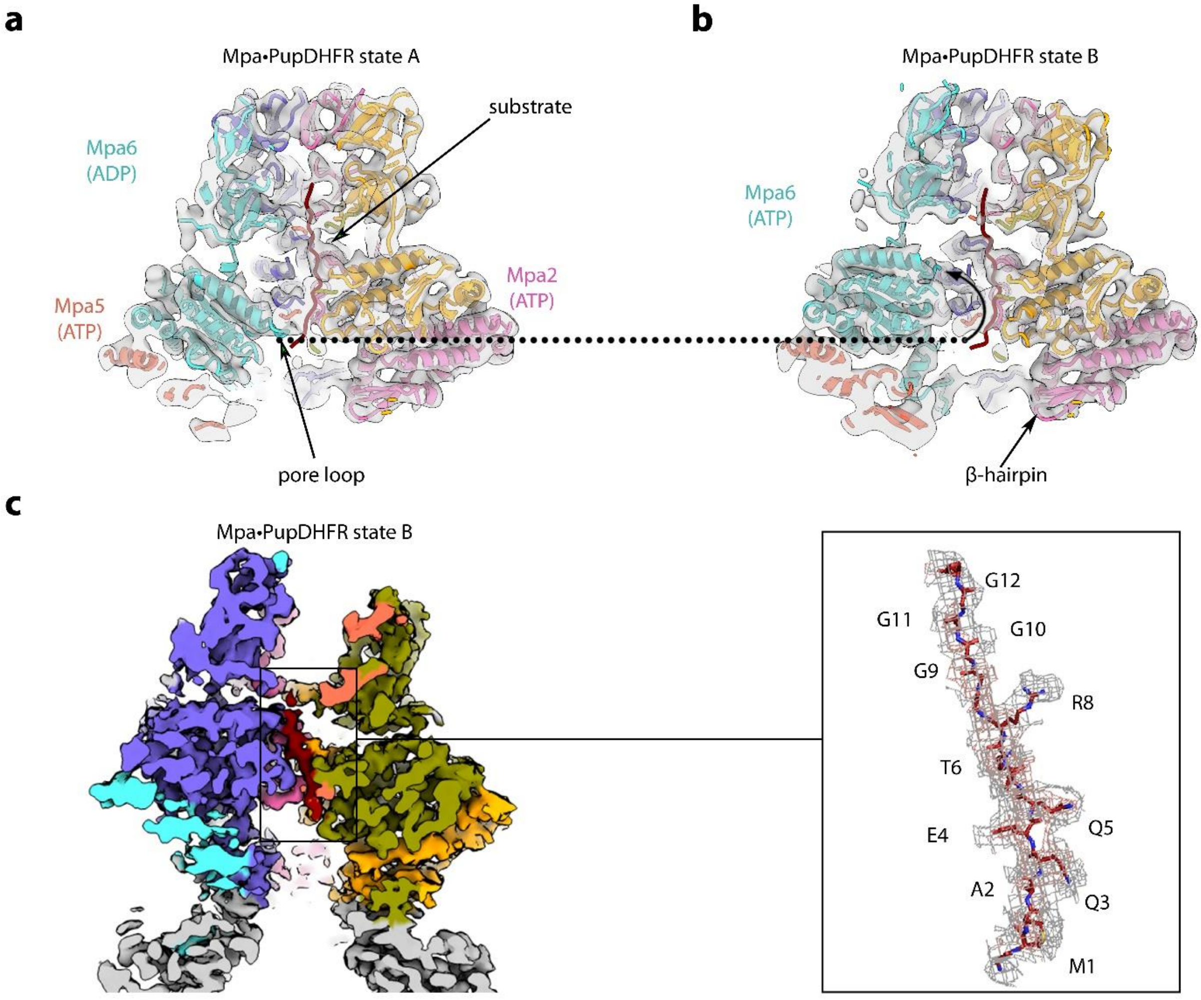

Supplementary Figure 7. Cross-section of substrate-engaged Mpa. a-b Cross-section of Mpa states A and B with cryo-EM maps shown as transparent surface and low-pass filtered to 6 Å resolution with underlying atomic coordinates shown as cartoon. Mpa β-hairpin, pore loop, and the substrate (maroon) are indicated with an arrow. The dashed line indicates the position of the pore loop of Mpa6 (cyan) in state A. The curved arrow indicates the conformational change of the pore loop in protomer 6 from state A to state B. **c**, Cross-section of the cryo-EM density of the 20S α-ring gate (grey) and Mpa (State B) engaged with

the N-terminal Pup segment inside the AAA chamber. EM density corresponding to the first 12 residues of Pup inside the Mpa ATPase chamber is shown with fitted atomic coordinates.

Supplementary Figure 8

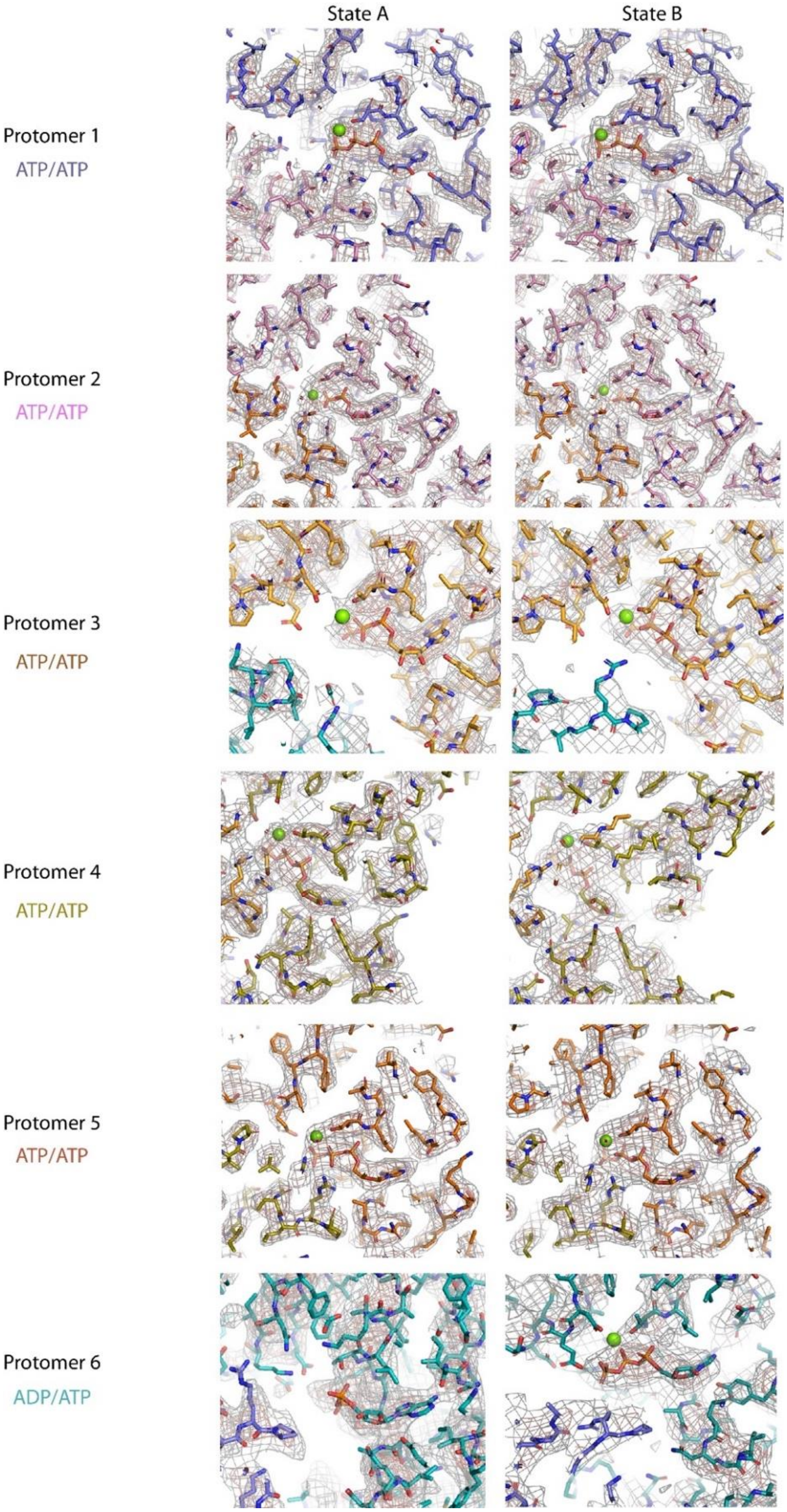

Supplementary Figure 8. Bound Nucleotides in Mpa states A and B. Close-up of the EM-densities shown as mesh with underlying atomic models shown as sticks. Identities of

the bound nucleotides in states A and B, respectively, and Mpa protomer number are indicated on the left side of the panels.

Supplementary Figure 9

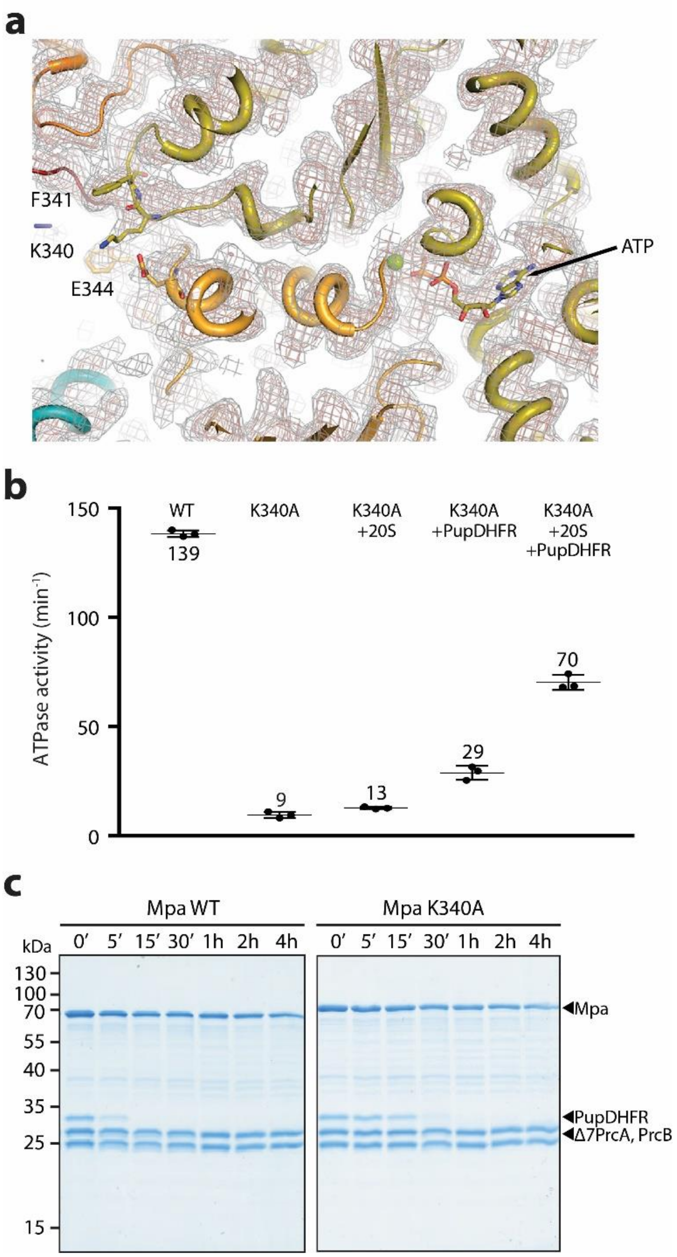

Supplementary Figure 9. Mpa pore loop 1 residue K340 can form a salt-bridge with a negatively charged residue (E344) from the neighboring protomer. a, Density at the interface between subunits illustrates the tentative interaction of K340 (protomer 4) with E344 (protomer 5). K340 of pore loop 1 can play a mechanistic role in the conformational cycle of loop movements coupled to the ATPase activity by forming a salt-bridge with E344 from the neighboring protomer. **b**, Binding of substrate and open-gate proteasome stimulates the Mpa

K340A ATPase activity to about half of the activity observed for wild type Mpa. The basal Mpa K340A ATPase activity is increased around 1.4-fold in the presence of the proteasome, 3.2-fold in the presence of PupDHFR and 7.8-fold in the presence of both. ATPase activity is indicated per Mpa hexamer. Error bars represent s.e. from three experiments. Data are presented as mean values ± SEM. Source Data are provided as a Source Data file. **c**, Proteasomal degradation of PupDHFR mediated by wild type Mpa (left) or Mpa K340A (right) was monitored by Coomassie-stained SDS-PAGE. The reaction was started with ATP and carried out at 37°C. Aliquots were taken at the indicated time points and analyzed by SDS-PAGE. Representatives of three individual experiments are shown. Source Data are provided as a Source Data file.

Supplementary Table 1. Cryo-EM data collection, refinement and validation statistics

	20S CP	Mpa-PupDHFR State A	Mpa-PupDHFR State B
**Data collection and processing**			
Nominal Magnification (pre-Gif)	105,000	105,000	105,000
Voltage (kV)	300	300	300
Electron exposure (e–/Å^2)	50	50	50
Defocus range (μm)	1.5 – 2.5	1.5 – 2.5	1.5 – 2.5
Pixel size (Å)	0.84	0.84	0.84
Binned Pixel size (Å)	1.22	1.22	1.22
Symmetry imposed	C1	C1	C1
Initial particle images (no.)	860,718	860,718	860,718
Final particle images (no.)	222,719	48,054	50,814
Map resolution (Å)	2.80	3.84	3.98
FSC threshold	0.143	0.143	0.143
Map resolution range (Å)	2.6 - 3.9	3.6 - 6.0	3.6 - 6.5
**Refinement**			
Initial model used (PDB code)	5LZP	5KWA	5KWA
Model resolution (Å)	2.9	4.0	4.2
FSC threshold	0.5	0.5	0.5
Map sharpening *B* factor (Å^2)	-66.51	-94.98	-111.8
Model composition			
Non-hydrogen atoms	46,636	22,194	22,286
Protein residues	6,192	2,817	2,828
Ligands	0	11	12
B factors (Å^2)			
Protein	51.89	88.83	91.13
Ligand	0	67.72	69.08
R.m.2. s. deviations			
Bond lengths (Å)	0.002	0.002	0.002
Bond angles (°)	0.390	0.518	0.524
Validation			
MolProbity score	1.20	1.92	1.89
Clash score	3.65	8.77	8.33
Poor rotamers (%)	1.14	1.89	1.96
Ramachandran plot			
Favored (%)	98.47	96.4	96.6
Allowed (%)	1.53	3.56	5.12
Outliers (%)	0.00	0.00	0.00

Supplementary Table 2. Oligonucleotide primers used in this study.
Overhangs for Gibson assembly are shown in lowercase letters.

Primer name	Sequence (5'-3')	Purpose
mpa-px-fw	ccggtgacgagcCTCCCGGCGTAATGCCTC	*mpa* KO generation
mpa-px-rv	cgactctgacggcagtttacCACGACGTGTGAGTGGTC	*mpa* KO generation
mpa-ds-fw	gttgccattgctgcaggcatcGGTCCGATCCGCAGGATC	*mpa* KO generation
mpa-ds-rv	cattacgccgggagGCTCGTCACCGGCAAG	*mpa* KO generation
p2NIL-fw3	GTAAACTGCCGTCAGAGTCG	*mpa* KO generation
clgr-ds-rv2	attcctaaccttttcgatccccCGTTCCGAGGCCAGCAG	*mpa* KO generation
dmpa-seq-fw2	GGACCGCCTTGCCTTGAATAG	*mpa* KO sequencing
dmpa-seq-rv	GAATCGCCGGTAGCGTTG	*mpa* KO sequencing
MK001_backbone_fw	TAAGAGCTCCGTCGACAAGC	pET20-His10-Trx-TEV-Pup amplification
MK001_backbone_rv	CTcTCCGCCCTTTTGAACGTATG	pET20-His10-Trx-TEV-Pup amplification
MK001_insert_fw	catacgttcaaaagggcggagagATGATCAGTCTGATTGCGGCG	*E.coli* DHFR gene amplification
MK001_insert_rv	gcttgtcgacggagctcttaTTACCGCCGCTCCAGAATC	*E.coli* DHFR gene amplification
rv006_007	GTAGGTGACCTCGAGGAA	pET20-Mpa HL^{Cg} and pMyNTint-Mpa HL^{Cg}-cloning
cglu_fw	ATCGATGGCTCCGTCGAGGTCATG TACTTCAAGG	pET20-Mpa HL^{Cg} and pMyNTint-Mp HL^{Cg}-cloning
110_k_fw	GCGTTCGTCGGGGAAAC	pET20-MpaK340A cloning
110+220_k_rv	GTTCAGCAGCTCGGG	pET20-MpaK340A cloning
3RA_fw	GAGTTGGCGgctAAGGGCATTGC	pET-Duet-1-Δ7PrcA-Δ57PrcB(H0-3RA) cloning
3RA_rv	GCTtgcCTCtgcCATCGCCTGCTC	pET-Duet-1-Δ7PrcA-Δ57PrcB(H0-3RA) cloning
