# Supplementary material for: Structural basis of prokaryotic ubiquitin-like protein engagement and translocation by the mycobacterial Mpa-proteasome complex
Source: Nat Commun. 2022 Jan 12;13:276. doi: 10.1038/s41467-021-27787-3 (PMC8755798; doi:10.1038/s41467-021-27787-3)
Supplement: Supplementary file 2 — Reporting Summary [file 41467_2021_27787_MOESM2_ESM.pdf]

nature portfolio

Corresponding author(s): Eilika Weber-Ban, Ahmad Jomaa

Last updated by author(s): Dec 2, 2021

Reporting Summary

Nature Portfolio wishes to improve the reproducibility of the work that we publish. This form provides structure for consistency and transparency in reporting. For further information on Nature Portfolio policies, see our Editorial Policies and the Editorial Policy Checklist.

Statistics

For all statistical analyses, confirm that the following items are present in the figure legend, table legend, main text, or Methods section.

n/a	Confirmed	
☐	☒	The exact sample size (*n*) for each experimental group/condition, given as a discrete number and unit of measurement
☐	☒	A statement on whether measurements were taken from distinct samples or whether the same sample was measured repeatedly
☒	☐	The statistical test(s) used AND whether they are one- or two-sided<br>*Only common tests should be described solely by name; describe more complex techniques in the Methods section.*
☒	☐	A description of all covariates tested
☒	☐	A description of any assumptions or corrections, such as tests of normality and adjustment for multiple comparisons
☐	☒	A full description of the statistical parameters including central tendency (e.g. means) or other basic estimates (e.g. regression coefficient) AND variation (e.g. standard deviation) or associated estimates of uncertainty (e.g. confidence intervals)
☒	☐	For null hypothesis testing, the test statistic (e.g. *F*, *t*, *r*) with confidence intervals, effect sizes, degrees of freedom and *P* value noted<br>*Give P values as exact values whenever suitable.*
☒	☐	For Bayesian analysis, information on the choice of priors and Markov chain Monte Carlo settings
☒	☐	For hierarchical and complex designs, identification of the appropriate level for tests and full reporting of outcomes
☒	☐	Estimates of effect sizes (e.g. Cohen's *d*, Pearson's *r*), indicating how they were calculated

Our web collection on statistics for biologists contains articles on many of the points above.

Software and code

Policy information about availability of computer code

Data collection	cryo EM data collection was done using ThermoFisher EPU2.0 software
Data analysis	GCTF v1.06, COOT 0.9.2, PHENIX 1.18.2, RELION3, CryoSPARC v.2, MotionCor2, UCSF ChimeraX 1.1, PyMOL 2.3.2, ESPript 3.0, GraphPad Prism 7.04, Origin 2018 (b.9.5.0.193), ClustalX2.1

For manuscripts utilizing custom algorithms or software that are central to the research but not yet described in published literature, software must be made available to editors and reviewers. We strongly encourage code deposition in a community repository (e.g. GitHub). See the Nature Portfolio guidelines for submitting code & software for further information.

Data

Policy information about availability of data

All manuscripts must include a data availability statement. This statement should provide the following information, where applicable:

- Accession codes, unique identifiers, or web links for publicly available datasets
- A description of any restrictions on data availability
- For clinical datasets or third party data, please ensure that the statement adheres to our policy

Protein structure data and the coordinates generated in this study have been deposited in the Protein Data Bank with PDB accession codes 7PX9 [https://doi.org/10.2210/pdb7PX9/pdb] (Substrate-engaged Mpa in state A- focused 3D refinement), 7PXA [https://doi.org/10.2210/pdb7PXA/pdb] (open-gate 20S CP - global 3D refinement), 7PXB [https://doi.org/10.2210/pdb7PXB/pdb] (Substrate-engaged Mpa in state B - focused 3D refinement), 7PXC [https://doi.org/10.2210/pdb7PXC/pdb] (Substrate-engaged Mpa in state A in complex with open-gate 20S CP - composite map) and 7PXD [https://doi.org/10.2210/pdb7PXD/pdb] (Substrate-engaged Mpa in state A in complex with open-gate 20S CP - composite map). Cryo-EM maps have been deposited with the Electron Microscopy Data Bank (EMDB) as EMDB-13694 (Substrate-engaged Mpa in state A- focused 3D refinement), -13695 (open-gate 20S CP - global 3D refinement), -13696 (Substrate-

engaged Mpa in state B - focused 3D refinement), -13697 (Substrate-engaged Mpa in state A in complex with open-gate 20S CP - composite map) and -13698 (Substrate-engaged Mpa in state B in complex with open-gate 20S CP - composite map). Source data are provided with this paper.

Field-specific reporting

Please select the one below that is the best fit for your research. If you are not sure, read the appropriate sections before making your selection.

☒ Life sciences ☐ Behavioural & social sciences ☐ Ecological, evolutionary & environmental sciences

For a reference copy of the document with all sections, see nature.com/documents/nr-reporting-summary-flat.pdf

Life sciences study design

All studies must disclose on these points even when the disclosure is negative.

Sample size	No statistical tests were used to predetermine sample sizes. Sample size per experiment and per strain/protein variant was one, because multiple repeats of the experiment were carried out. Numbers of repeats for in vivo RecA degradation, in vitro PupDHFR degradation and ATPase assay were chosen based on established practice in the field and were sufficient as the results were reproducible.
Data exclusions	No data were excluded in biochemical experiments. During cryo-EM analysis, picked coordinates which did not correspond to protein particles were discarded after 2D classification. Particles which did not correspond to the Mpa-proteasome complex were discarded following global and focused 3D classification. Particles belonging to those Mpa 3D classes that produced low resolution maps during refinement were also excluded. See Supplementary Figure 1 for more details.
Replication	In vivo RecA degradation experiments were repeated 3 times individually for each strain starting from the single colony. In vitro PupDHFR degradation assays were repeated 3 times individually for every Mpa and proteasome variant. ATPase assay was repeated 3 times individually for every Mpa variant as well as for Mpa K340 in the presence of 20S, substrate and both.
Randomization	Not applicable to our study, since no treatment groups were handled in the experiments
Blinding	Not applicable to our study. No populations were preassigned to groups so blinding was not relevant. Investigators need to be able to identify the bacterial strains in the culture flasks at any given time due to safety regulations.

Reporting for specific materials, systems and methods

We require information from authors about some types of materials, experimental systems and methods used in many studies. Here, indicate whether each material, system or method listed is relevant to your study. If you are not sure if a list item applies to your research, read the appropriate section before selecting a response.

Materials & experimental systems

n/a	Involved in the study	
☐	☒	Antibodies
☒	☐	Eukaryotic cell lines
☒	☐	Palaeontology and archaeology
☒	☐	Animals and other organisms
☒	☐	Human research participants
☒	☐	Clinical data
☒	☐	Dual use research of concern

Methods

n/a	Involved in the study	
☒	☐	ChIP-seq
☒	☐	Flow cytometry
☒	☐	MRI-based neuroimaging

Antibodies

Antibodies used	α-RpoB (E. coli) monoclonal (mouse), BioLegend clone #8RB13 Cat. #663903<br>α-RecA (E. coli) monoclonal (mouse), LabForce clone #ARM414 Acris/OriGene #AM26666AF-N epitope Glu233-Lys256<br>α-mouse igG polyclonal (goat) Promega #W4021
Validation	α-RpoB (E. coli), α-RecA (E. coli) and α-mouse IgG antibodies were validated by the manufactures via western blot analysis as outlined on their websites.
